# Genomic characterization of malonate positive *Cronobacter sakazakii* serotype O:2, sequence type 64 strains, isolated from clinical, food, and environment samples

**DOI:** 10.1186/s13099-018-0238-9

**Published:** 2018-03-10

**Authors:** Gopal R. Gopinath, Hannah R. Chase, Jayanthi Gangiredla, Athmanya Eshwar, Hyein Jang, Isha Patel, Flavia Negrete, Samantha Finkelstein, Eunbi Park, TaeJung Chung, YeonJoo Yoo, JungHa Woo, YouYoung Lee, Jihyeon Park, Hyerim Choi, Seungeun Jeong, Soyoung Jun, Mijeong Kim, Chaeyoon Lee, HyeJin Jeong, Séamus Fanning, Roger Stephan, Carol Iversen, Felix Reich, Günter Klein, Angelika Lehner, Ben D. Tall

**Affiliations:** 1 Center of Food Safety and Applied Nutrition, U. S. Food and Drug Administration, Laurel, MD 20708 USA; 2 Institute for Food Safety and Hygiene, University of Zurich, Zurich, Switzerland; 3 UCD Centre for Food Safety, School of Public Health, Physiotherapy & Population Science, University College, Dublin & WHO Collaborating Centre for Cronobacter, Belfield, Dublin 4, Ireland; 4 Institute for Food Quality and Safety, University of Veterinary Medicine Hannover, Bischofsholer Damm 15, 30173 Hannover, Germany

**Keywords:** Malonate utilization in *C. sakazakii*, DNA microarray, Whole genome sequencing, Phylogenetic analysis

## Abstract

**Background:**

Malonate utilization, an important differential trait, well recognized as being possessed by six of the seven *Cronobacter* species is thought to be largely absent in *Cronobacter sakazakii* (Csak). The current study provides experimental evidence that confirms the presence of a malonate utilization operon in 24 strains of sequence type (ST) 64, obtained from Europe, Middle East, China, and USA; it offers explanations regarding the genomic diversity and phylogenetic relatedness among these strains, and that of other *C. sakazakii* strains.

**Results:**

In this study, the presence of a malonate utilization operon in these strains was initially identified by DNA microarray analysis (MA) out of a pool of 347 strains obtained from various surveillance studies involving clinical, spices, milk powder sources and powdered infant formula production facilities in Ireland and Germany, and dried dairy powder manufacturing facilities in the USA. All ST64 *C. sakazakii* strains tested could utilize malonate. Zebrafish embryo infection studies showed that *C. sakazakii* ST64 strains are as virulent as other *Cronobacter* species. Parallel whole genome sequencing (WGS) and MA showed that the strains phylogenetically grouped as a separate clade among the Csak species cluster. Additionally, these strains possessed the Csak O:2 serotype. The nine-gene, ~ 7.7 kbp malonate utilization operon was located in these strains between two conserved flanking genes, *gyrB* and *katG.* Plasmidotyping results showed that these strains possessed the virulence plasmid pESA3, but in contrast to the USA ST64 Csak strains, ST64 Csak strains isolated from sources in Europe and the Middle East, did not possess the type six secretion system effector *vgrG* gene.

**Conclusions:**

Until this investigation, the presence of malonate-positive Csak strains, which are associated with foods and clinical cases, was under appreciated. If this trait was used solely to identify *Cronobacter* strains, many strains would likely be misidentified. Parallel WGS and MA were useful in characterizing the total genome content of these Csak O:2, ST64, malonate-positive strains and further provides an understanding of their phylogenetic relatedness among other virulent *C. sakazakii* strains.

**Electronic supplementary material:**

The online version of this article (10.1186/s13099-018-0238-9) contains supplementary material, which is available to authorized users.

## Background

*Cronobacter* species are Gram-negative bacteria which can cause severe infantile septicemia, meningitis, and necrotizing enterocolitis and pose a serious threat to neonates and underweight infants [1, 2]. *Cronobacter* species can also cause infections in adults with a high percentage of infections presenting as septicemia, pneumonia, wound, and urinary tract infections [3–5]. The genus *Cronobacter* has seven species: *Cronobacter sakazakii*, *Cronobacter malonaticus*, *Cronobacter turicensis*, *Cronobacter universalis*, *Cronobacter dublinensis*, *Cronobacter muytjensii*, and *Cronobacter condimenti* [6, 7]. Since the discovery of *C. condimenti* in 2012, there has been no epidemiological evidence associating this species with human infections and thus was considered to be avirulent. Recently Eshwar et al. [8] performed infection studies where zebrafish embryos were exposed to *C. condimenti* and found that *C. condimenti* strain LMG 26250^T^ caused an 80% mortality rate within 4 days post infection suggesting that it is as virulent as other *Cronobacter* species. However, *C. sakazakii*, *C. malonaticus*, and *C. turicensis* are currently considered to be the primary pathogenic species found to cause the majority of illnesses [9]. Infantile *Cronobacter* infections have often been linked to the consumption of reconstituted, temperature-abused, intrinsically or extrinsically contaminated powdered infant formulas (PIF). Because PIF is not manufactured as a sterile product, it poses a significant consumer risk should contaminated lots be prepared and handled inappropriately. Subsequently this led to the publishing of guidelines for proper PIF preparation (http://www.who.int/foodsafety/publications/micro/PIF_Bottle_en.pdf) by the World Health Organization. Notably, Jason [10] reported that 8% (7/82) of infected infants studied during 2004–2010 presented with invasive disease (defined as a culture-positive, confirmed case of septicemia or meningitis) and consumed breast milk without any PIF or human milk fortifier supplementation prior to onset of illness. Freidemann and Bowen have reported similar findings [11, 12]. Aside from contaminated PIF, sources of these infections in both infants and adults have been elusive. Additionally, *Cronobacter* species can be detected within other dried foods, ready to eat foods, and food production environments, such as in dried food manufacturing facilities, posing a risk to susceptible consumers [11, 13]. Thus, it is important that the food manufacturing and public health communities continue surveillance efforts to find the presence of these organisms in food products and within food processing environments.

Malonate is thought to be produced both in root tissues and leaves of plants [14]. Malonate utilization has been a trait well recognized as being possessed by six of the seven *Cronobacter* species, excluding *C. sakazakii*. However, Iversen et al. [6] reported that a small number (< 5%) of *C. sakazakii* strains can also utilize malonate. Until the present investigation, this finding was largely overlooked and under-appreciated. Representative malonate utilization operon alleles were first observed in a group of *C. sakazakii* strains obtained from a surveillance assignment of USA dairy powder manufacturers in 2014 using a previously described custom-designed, novel pan genomic DNA microarray [15]. The microarray contains 50 pan-genomically conserved malonate operon alleles which are represented on the array and primarily come from the genomes of *C. turicensis* and *C. malonaticus*. These genes are located in Genome Region (GR) GR34 of *C. malonaticus* type strain LMG23826^T^, as described by Grim et al. [16]. GR34 encodes for the components of enzymes and proteins involved in the decarboxylation of malonate, and include a malonate utilization transcriptional regulator *mdcR*, a malonate transporter gene, *mdcF*, and the gene *mdcE*, which encodes for a stabilization protein. MdcE is thought to stabilize MdcF with the beta chain of the acetyl-coenzyme-A carboxyl transferase. Malonate decarboxylase in *C. malonaticus* comprises the oligomerization of alpha, delta, beta, and gamma protein subunits and is encoded by four genes, *mdcADBC*. Finally, this gene cluster also contains genes encoding for a 2-(5′-triphosphoribosyl)-3′-dephosphocoenzyme-A synthase (*ybdT*), malonyl CoA acyl carrier protein transacylase (*fabD*) and a phosphoribosyl-dephospho-CoA transferase (*mdcG*), which are thought to stabilize the coenzyme-A complex [15, 16].

The under appreciation of malonate-positive *C. sakazakii* strains that are associated with foods suggests that possible misidentification when relying on phenotypic identification schemes alone may occur, hampering correct species identification for epidemiological purposes. Proper species identification of *Cronobacter* is imperative given the recent proposals by Jason [10], Friedemann [11], and Farmer [17] to include this organism as a member of the Centers for Disease Control and Prevention’s and the Council of State and Territorial Epidemiologists’ notifiable disease list.

Microarray analysis (MA) and subsequent whole genome sequencing (WGS) analysis of *C. sakazakii* strain CDC 1121–73, a serotype Csak O:2, sequence type (ST) 64 clinical strain obtained in 1973, showed that this strain possessed the alleles of an entire malonate utilization operon [18]. With this uncharacteristic finding, further microarray interrogation of other *C. sakazakii* strains revealed that 22 additional *C. sakazakii* strains from spice, milk powder, dairy powder and PIF manufacturing facilities in the USA, Middle East, and Europe possessed the entire operon as well.

The Food and Drug Administration’s (FDA) and its global food safety partners’ capacity to protect the public rests on the ability to identify and characterize foodborne pathogens quickly and accurately so that circulating contaminated foods can be rapidly removed from commerce. In addition to gaining an understanding of the pathogenicity of the species, this study also assessed WGS as a method for characterization of *Cronobacter* strains, a method which is hoped to one day be adapted clinically for detection and treatment purposes. The main purpose of this study was to analyze the genomic content of these recently recognized malonate-positive *C. sakazakii* strains that originated from clinical, milk powder, spice, and powdered infant formula and dairy powder manufacturing environments using next generation sequence tools such as DNA microarray and WGS. We hope that the knowledge reported here regarding these organisms will add to the surmounting body of genomic information about this life-threatening foodborne and noted public health pathogen so that relevant molecular clinical and food diagnostic assays may be further developed.

## Methods

### Bacterial strains

The *Cronobacter* strains analyzed in this study, with corresponding metadata, are listed in Table 1. These strains represent isolates acquired from foods, environmental and clinical sources and were obtained from multiple and diverse geographical areas and were selected out of a pool of 347 strains obtained from various surveillance studies [19–22]. Identity of *Cronobacter* species was based on the *Cronobacter* classification scheme as proposed by Iverson et al. [6] and Joseph et al. [7]. Additionally, all strains tested positive for the 350 bp zinc metalloprotease (*zpx*) genus-level gene [19]. Species identity was also established using the species-specific *rpoβ* PCR assay as noted by Stoop et al. [23] and Lehner et al. [24], and the *cgcA* species-specific PCR assay as described by Carter et al. [20]. These strains were serotyped according to the typing scheme proposed by Yan et al. [21]. The isolates were also subjected to RepF1B plasmidotyping as described by Franco et al. [22]. Multiple locus sequence typing (MLST, ST) of the strains was performed either by uploading genome FASTA sequences to the *Cronobacter* MLST website (http://pubmlst.org/cronobacter/) or by performing the PCR reactions according to the procedure described by Baldwin et al. [25] and Jolley and Maiden [26]. Prior to submission to the *Cronobacter* MLST website, PCR amplicons were first purified using the Qiagen PCR purification kit (Qiagen, Inc. Germantown, MD), and submitted to Macrogen, Inc. (Rockville, MD) for sequencing. Where noted, we have confirmed the STs of the strains reported in Table 1 through using their WGS assemblies and the MLST website listed above. The genome sequence for *C. sakazakii* strain Cro2819A3 as reported by Zheng et al. [27] was obtained from the National Center for Biotechnology Information (NCBI) under accession #: NZ_MBSC00000000. Malonate utilization was assessed for all strains (except for *C. sakazakii* strain Cro2819A3) and controls using Malonate broth (Ewing’s Modification, Thermo Fisher, Inc. Grand Island, NY). These organisms could utilize sodium malonate as a carbon source and ammonium sulfate as a nitrogen source which produced an alkaline reaction (> pH 7.4) leading to a color change of the bromothymol blue (bromothymol sulfone phthalein) indicator dye from green to blue as described by Leifson [28] and Ewing et al. [29].

**Table 1:** General features, sources, malonate reaction, and genomic attributes of the strains used in this study

Strain	Source (isolation date)	Malonate utilized^a^	Serotype	Sequence type	Clonal complex	Draft genome size (bp)	G+C content (%)	Contigs	CDS	NCBI accession no.
7405-71-1A (Comp 11)	Environmental, compliance activity (2014), USA	(+)	Csak O:2	64	64	4,432,172	56.74	124	4104	NHQL00000000
7405-68-2 (Comp 19)	Environmental, compliance activity (2014), USA	(+)	Csak O:2	64	64	4,537,591	56.85	121	4205	NHQM00000000
7405-71-1B (Comp 20)	Environmental, compliance activity (2014), USA	(+)	Csak O:2	64	64	4,564,009	56.77	190	4228	NEXY00000000
867009 DFI106-1 (Comp 45)	Environmental, compliance activity (2014), USA	(+)	Csak O:2	64	64	4,731,392	56.8	117	4213	NEXZ00000000
867009 RF106-1 (Comp 46)	Environmental, compliance activity (2014), USA	(+)	Csak O:2	64	64	4,728,640	56.77	107	4407	NEYA00000000
7405-80-2 (Comp 49)	Environmental, compliance activity (2014), USA	(+)	Csak O:2	64	64	4,498,706	56.9	108	4159	NEYB00000000
7405-68-4 (Comp 53)	Environmental, compliance activity (2014), USA	(+)	Csak O:2	64	64	4,545,852	56.8	101	4,177	NEYC00000000
7405-75-1 (Comp 54)	Environmental, compliance activity (2014), USA	(+)	Csak O:2	64	64	4,540,274	57	135	4210	NEYD00000000
7405-75-2 (Comp 57)	Environmental, compliance activity (2014), USA	(+)	Csak O:2	64	64	4,538,462	56.8	101	4200	NEYE00000000
0210ELJ 72-1 (Comp 59)	Environmental, compliance activity (2014), USA	(+)	Csak O:2	64	64	4,649,928	56.5	173	4310	NEYF00000000
CDC 1121-73^c^	Human, clinical Bronchial. wash (1973), USA	(+)	Csak O:2	64	64	4,443,589	57	238	4112	MCOD00000000
GK 1025^c^	Environmental, PIF manufacturing facility (2015), Germany	(+)	Csak O:2	64	64	4,599,266	56.7	94	4295	MCOE00000000
GK 1026	Environmental, PIF manufacturing facility (2015), Germany	(+)	Csak O:2	64	64	4,603,121	56.73	126	4298	NEYG00000000
GK 1027	Environmental, PIF manufacturing facility (2015), Germany	(+)	Csak O:2	64	64	4,603,249	56.7	34	4304	NHQN00000000
GK 1028	Environmental, PIF manufacturing facility (2015), Germany	(+)	Csak O:2	ND	ND	ND	ND	ND	ND	Not Yet Submitted
GK 1029	Environmental, PIF manufacturing facility (2015), Germany	(+)	Csak O:2	64	64	4,589,912	56.7	95	4292	NHQO00000000
GK 1030	Environmental, PIF manufacturing facility (2015), Germany	(+)	Csak O:2	64	64	4,601,082	56.7	37	4286	NHQP00000000
GK 1034	Environmental, PIF manufacturing facility (2015), Germany	(+)	Csak O:2	64	64	4,604,244	56.7	29	4296	NHQQ00000000
GK 1035	Environmental, PIF manufacturing facility (2015), Germany	(+)	Csak O:2	64	64	4,597,230	56.7	50	4282	NHQR00000000
GK 1326	Environmental, PIF manufacturing facility (2015), Germany	(+)	Csak O:2	64	64	4,423,544	56.73	153	4096	NEYH00000000
E772	Milk powder, France	(+)	Csak O:2	64	64	4,537,187	56.8	351	4163	NHQS00000000
H169/1/16	Environmental, PIF manufacturing facility (2012), Switz	(+)	Csak O:2	64	64	4,474,959	57	32	4139	NHTV00000000
Jor172	Food, spices, Jordan	(+)	Csak O:2	64	64	4,330,450	57	25	4000	NCWD00000000
Cro2819A3^c^	Mushroom (2017), China	ND	ND	64	64	4,725, 000	57.2	168	3994	NZMBSC00000000
*C. universalis* NCTC9529^T^	Water, (1956), UK	(+)	Cuni O:1	54	Unknown^b^	4,388,239	55.79	16	3977	CAKX00000000
*C. condimenti* LMG26250^T^	Fermented spiced sausage, (2010), Slovakia	(+)	Unknown	98	Unknown^b^	4,480,620	55.8	155	4169	CAKW00000000
*C. dublinensis* LMG23823^T^	Environmental, milk powder manufacturing plant (2004), Ireland	(+)	Cdub O:1	106	Unknown^b^	4,644,913	56.16	41	4172	CP012266
*C. malonaticus* LMG23826^T^	Clinical, breast abscess (1977), USA	(+)	Cmal O:2	7	7	4,419,871	54.9	69	4041	CP013940
*C. turicensis* LMG23827^T^	Clinical, blood (2005), Switzerland	(+)	Ctur O:1	19	24	4,599,092	57.2	1	4296	FN543093
*C. muytjensii* 51329^T^	Unknown, USA	(+)	Cmuy O:2	81	81	4,355,922	56.17	32	3973	CP012268
*C. sakazakii* BAA-894	Infant formula (2001), USA	(−)	Csak O:1	1	1	4,530,777	56.7		4211	CP000783

*Cronobacter* species identities were established using the species-specific *rpoβ* PCR assay as described by Stoop et al. [23] and Lehner et al. [24], and the *cgcA* species-specific PCR assay as described by Carter et al. [20]. Moreover, serotyping was assigned using the molecular-based serogrouping scheme described by Yan et al. [21], the results of which confirmed results of the* rpoβ*- and* cgcA*-based PCR species identification assays for each strain

*ND* not determined

^a^Malonate utilization was performed using Ewing Modified Malonate Broth according to the original assay described by Leifson [28] and modified by Ewing et al. [29]. Results were summarized after 48 h incubation at 37 °C

^b^No clonal complex was found for this strain in the *Cronobacter* MLST database

^c^*C. sakazakii* strain Cro2819A3 was submitted to NCBI by Zeng et al. [27], and strains 1025 and 1121-73 were previously reported by Chase et al. [18]

### DNA extraction and genome sequencing and analysis

Frozen bacterial cultures were stored at − 80 °C in Trypticase soy broth (BBL, Cockeysville, MD) supplemented with 1% NaCl (TSBS) and 50% glycerol and were streaked onto plates containing *Enterobacter sakazakii* Chromogenic Plating Medium (R&F Products; Downers Grove, IL) and incubated overnight at 37 °C. Typical *Cronobacter*-like colonies were chosen to inoculate duplicate TSBS broth cultures (5 ml) which were incubated at 37 °C, shaking at 150 rpm for 18 h. Bacterial DNA was extracted and purified using Qiagen’s Qiacube technology (QIAGEN Sciences; Germantown, MD) according to the manufacturer’s instructions. The concentrations of the DNA samples were determined using a NanoDrop spectrophotomer (Thermo Fisher Scientific; Wilmington, DE). Typically, the DNA samples possessed a DNA concentration of between 10 and 60 ng/μl. For WGS analysis of the strains, a more precise measurement of the concentration of these DNAs was then determined using a Qubit Fluorometric spectrophotometer (Life Technologies, Thermo Fisher Scientific; Wilmington, DE) for quantitation, using one of the DNA sample replicates. DNA samples were diluted with deionized water to a final concentration of 0.2 ng/μ1. Whole-genome sequencing was performed using a MiSeq benchtop sequencer (Illumina, San Diego, CA, USA), utilizing either 500 or 600 cycles of paired-end reads (Illumina). FASTQ datasets were de novo assembled with CLC Genomics Workbench version 7.0 (CLC bio, Aarhus, Denmark). The paired end libraries were generated and sequenced in conjunction with the Nextera XT DNA Sample Preparation Guide on the Illumina Miseq instrument (Illumina; San Diego, CA). Sequence data for each strain was uploaded onto the Rapid Annotation Subsystems Technology (RAST) server for annotation [30]. For microarray analysis, the duplicated purified DNA samples were further concentrated using an Amicon Ultracel-30 membrane filter (30,000 molecular weight cutoff, 0.5 ml, Millipore Corp. Billerica, MA) to a final volume of approximately 10–25 µl. Comparative genomics and phylogenetic analysis were carried out using Geneious (http://www.geneious.com), LASTZ [31] implementation on Geneious, and MEGA 7 suite [32]. Local BLAST+ analysis was carried out wherever necessary. Whole genome SNP analysis was carried out using kSNP3 software [33].

### Microarray design, hybridization, and analysis

The DNA microarray used in this study was an Affymetrix custom array (Affymetrix design number: FDACRONOa520845F) which utilizes the whole genome sequences of 15 *Cronobacter* strains, as well as 18 plasmids. These 15 strains encompassed all proposed species of *Cronobacter*. This was the same microarray described previously by Tall et al. [15]. Genomic DNA was hybridized, washed in the Affymetrix FS-450 fluidics station, and scanned on the Affymetrix GeneChip^®^ Scanner 3000 (AGCC software) as described by Tall et al. [15, 34], Yan et al. [13], Chase et al. [35], and Kothary et al. [36]. All reagents for hybridizing, staining and washing were made in conjunction with the Affymetrix GeneChip^®^ Expression Analysis Technical Manual [37]. For each gene represented on the microarray, associated probe set intensities were summarized using the Robust MultiArray Averaging (RMA) function in the Affymetrix package of R-Bioconductor as described by Bolstad et al. [38]. RMA summarization, normalization, and polishing was done on the data received and final probe set values were determined as described by Tall et al. [15]. Gene differences were determined and phylogenetic trees were created using the SplitsTree 4 neighbor net joining method.

### ZebraFish infection studies

Husbandry, breeding and microinjection of approx. 50 CFU of bacteria into the yolk sac of 2-day post fertilization albino Zebrafish (*Danio rerio*) was maintained following the original procedure described in the study by Fehr et al. [39] and later by Eshwar et al. [8]. A total of thirty embryos (10 × 3) were injected per individual experiment (i.e. per strain). This research was conducted with approval (NO 216/2012) from the Veterinary Office, Public Health Department, Canton of Zurich (Switzerland) allowing experiments with embryos and larvae older than 120 dpf.

### Nucleotide sequence accession numbers

Nucleotide sequences from this study were deposited into GenBank under accession numbers identified in Table 1. The sequences of the strains were also released to the public by submission to the NCBI under the *Cronobacter* GenomeTrakr Project: FDA-CFSAN bioproject: PRJNA258403 as part of the FDA’s Center for Food Safety and Applied Nutrition (CFSAN) surveillance project for rapid detection of foodborne pathogens causing illnesses or outbreaks.

## Results and discussion

### Identities and general features of malonate-positive, ST64 *C. sakazakii* strains

Twenty-three *C. sakazakii* strains were obtained from surveillance studies of dried milk powder, clinical, spice, and environmental samples taken from retail facilities, cheese and milk protein and powdered infant formula manufacturing facilities located in the USA, Middle East, and Europe by various investigators [13, 15, 19–22, 35]. General features of these strains, sources, malonate utilization, and genomic information of the strains are shown in Table 1. All strains were identified as *C. sakazakii* using the species-specific *rpoβ* PCR assays as described by Stoop et al. [23] and Lehner et al. [24], and the *cgcA* species-specific PCR assay as described by Carter et al. [20]. Moreover, all strains possessed the Csak O:2 serotype as defined by the protocol described by Yan et al. [21], confirming the *rpoβ*- and *cgcA*-based PCR species identity for each strain. The serotype and malonate utilization of *C. sakazakii* strain Cro2819A3 could not be assessed. Additionally, all strains were PCR-positive for *zpx*, a genus-specific target encoding for a zinc-containing metalloprotease gene [19]. All strains were identified as *C. sakazakii* ST64 using the *Cronobacter* MLST database (http://pubmlst.org/cronobacter/) [25, 26].

We had recently described the genomes of two ST64 strains CDC 1121–3 and GK1025 from our collection, which were used as the quintessential strains in this study [18]. The remaining 21 strains were sequenced and annotated as described earlier. As mentioned earlier, the genome of *C. sakazakii* strain Cro2819A3 was obtained from NCBI. The draft genomes sizes (Table 1), ranged from 4.3 to 4.7 MB, and the G+C% content ranged from 54.9 to 57.7. Around 4000–4407 DNA coding sequences were identified among the strains, sharing similar genomic features to the two earlier clinical and PIF production plant strains [18] and other genomes described for *C. sakazakii* [22, 40–42].

### Malonate utilization among *C. sakazakii* O:2, ST64 strains compared to other *Cronobacter* strains

Phenotypically, all tested ST64 *C. sakazakii* strains utilized malonate (shown in Table 1). Control strains included to demonstrate malonate utilization were: *C. universalis*, NCTC 9529^T^, *C. condimenti* LMG23826^T^, *C. malonaticus* LMG23826^T^, *C. turicensis* LMG23827^T^, *C. muytjensii* 51329^T^, and *C. dublinensis* subspp. *dublinensis* LMG23823^T^. *C. sakazakii* strain BAA-894 was used as a negative control and as expected, was found to be malonate negative.

### Characterization of RepFIB plasmids and pESA3-specific gene targets among *C. sakazakii* O:2, ST64 strains compared to other *Cronobacter* strains

Characterization of the strains for the presence of the common virulence plasmids, pESA3- and pSP291-1-like plasmids was performed. These plasmids share a high degree of sequence homology [22, 40, 41, 43], and they harbor a common incompatibility class IncF1B replicon-*repA* and two iron (III) acquisition systems-*eitCBAD* (ABC heme transporter) and *iucABCD/iutA* (Cronobactin, a hydroxamate-type, aerobactin-like siderophore). Targeting the homologous *repA* gene and the two iron acquisition system gene clusters (*eitA*, and *iucC* genes representing each gene cluster), PCR analysis showed that all strains possessed *repA*, *eitA*, and *iucC* genes, suggesting that the common virulence plasmids (pESA3, pSP291-1) were harbored by these strains (Table 2). Further PCR analysis of the strains showed that they all possessed the *Cronobacter* plasminogen activator gene, *cpa*. Interestingly, only the USA dairy environmental strains possess most of the type six secretion system gene cluster (T6SS) genes, e.g., T6SSIntL, *vgrG*, and T6SSRend targets: the strains which were isolated from the German and Swiss PIF manufacturing facilities, the milk powder sample from France and the spice strain from Jordan lacked *vgrG*. All strains lacked both the T6SS IntR and filamentous hemagglutinin (FHA) *fhaB* targets, but possessed the conserved flanking regions of FHA. These results suggest that the genomic region encompassing the T6SS may be undergoing microevolution similar to what Riccobono et al. has described for enteropathogenic *E. coli* [44] and what Franco et al. [22] and Yan et al. [13] had previously reported.

**Table 2:** Plasmidotyping results of strains used in this study

Strain	Plasmidotyping results by PCR analysis according to Franco et al. [22]																
	pESA3/p CTU1 incFIB	*eit*	*iuc*	*Cdiuc*	*cpa*	*Δcpa*	ΔT6SS	T6SS IntL	*vgrG*	T6SS R end	T6SS IntR	*Δfha*	*fha*	*dfha*	pESA2 IncF2	pCTU3 H1	*zpx*
7405-71-1A (Comp 11)	(+)	(+)	(+)	ND	(+)	(–)	ND	(+)	(+)	(+)	(–)	(+)	(–)	ND	(–)	(–)	(+)
7405-68-2 (Comp 19)	(+)	(+)	(+)	ND	(+)	(–)	ND	(+)	(+)	(+)	(–)	(+)	(–)	ND	(–)	(–)	(+)
7405-71-1B (Comp 20)	(+)	(+)	(+)	ND	(+)	(–)	ND	(+)	(+)	(+)	(–)	(+)	(–)	ND	(–)	(–)	(+)
867009 DFI106-1 (Comp 45)	(+)	(+)	(+)	ND	(+)	(–)	ND	(+)	(+)	(+)	(–)	(+)	(–)	ND	(–)	(–)	(+)
867009 RF106-1 (Comp 46)	(+)	(+)	(+)	ND	(+)	(–)	ND	(+)	(+)	(+)	(–)	(+)	(–)	ND	(–)	(–)	(+)
7405-80-2 (Comp 49)	(+)	(+)	(+)	ND	(+)	(–)	ND	(+)	(+)	(+)	(–)	(+)	(–)	ND	(–)	(–)	(+)
7405-68-4 (Comp 53)	(+)	(+)	(+)	ND	(+)	(–)	ND	(+)	(+)	(+)	(–)	(+)	(–)	ND	(–)	(–)	(+)
7405-75-1 (Comp 54)	(+)	(+)	(+)	ND	(+)	(–)	ND	(+)	(+)	(+)	(–)	(+)	(–)	ND	(–)	(–)	(+)
844916-23 (Comp 55)	(+)	(+)	(+)	ND	(+)	(–)	ND	(+)	(+)	(+)	(–)	(+)	(–)	ND	(–)	(–)	(+)
7405-75-2 (Comp 57)	(+)	(+)	(+)	ND	(+)	(–)	ND	(+)	(+)	(+)	(–)	(+)	(–)	ND	(–)	(–)	(+)
0210ELJ 72-1 (Comp 59)	(+)	(+)	(+)	ND	(+)	(–)	ND	(+)	(+)	(+)	(–)	(+)	(–)	ND	(–)	(+)	(+)
CDC 1121-73	(+)	(+)	(+)	(–)	(+)	(–)	(–)	(+)	(+)	(+)	(–)	(+)	(–)	(–)	ND	ND	(+)
GK 1025*	(+)	(+)	(+)	ND	(+)	(–)	ND	(+)	(–)	(+)	(–)	(+)	ND	ND	(+)	(+)	(+)
GK 1026*	(+)	(+)	(+)	ND	(+)	(–)	ND	(+)	(–)	(+)	(–)	(+)	ND	ND	(–)	(–)	(+)
GK 1027	(+)	(+)	(+)	ND	(+)	(–)	ND	(+)	(–)	(+)	(–)	(+)	ND	ND	(–)	(–)	(+)
GK 1028	(+)	(+)	(+)	ND	(+)	(–)	ND	(+)	(–)	(+)	(–)	(+)	ND	ND	(–)	(–)	(+)
GK 1029	(+)	(+)	(+)	ND	(+)	(–)	ND	(+)	(–)	(+)	(–)	(+)	ND	ND	(–)	(–)	(+)
GK 1030	(+)	(+)	(+)	ND	(+)	(–)	ND	(+)	(–)	(+)	(–)	(+)	ND	ND	(–)	(–)	(+)
GK 1034	(+)	(+)	(+)	ND	(+)	(–)	ND	(+)	(–)	(+)	(–)	(+)	ND	ND	(–)	(–)	(+)
GK 1035	(+)	(+)	(+)	ND	(+)	(–)	ND	(+)	(–)	(+)	(–)	(+)	ND	ND	(–)	(–)	(+)
GK 1326	(+)	(+)	(+)	ND	(+)	(–)	ND	(+)	(–)	(–)	(–)	(+)	ND	ND	(–)	(–)	(+)
E722	(+)	(+)	(+)	(–)	(+)	(–)	(–)	(+)	(–)	(+)	(–)	(+)	(–)	(–)	(–)	(–)	(+)
H169/1/16	(+)	(+)	(+)	ND	(+)	ND	ND	(+)	(–)	(+)	(–)	(+)	(–)	ND	(–)	(–)	ND
Jor172	(+)	(+)	(+)	(–)	(+)	(–)	(–)	(+)	(–)	(–)	(–)	(+)	(–)	(–)	(–)	(–)	(+)
*C. universalis* NCTC9529^T^	(+)	(+)	(+)	(–)	(+)	(–)	(+)	(–)	(–)	(–)	(–)	(–)	(+)	(–)	(–)	(–)	(+)
*C. condimenti* LMG26250^T^	(+)	(+)	(+)	(+)	(–)	(–)	(–)	(–)	(–)	(–)	(–)	(–)	(–)	(–)	ND	ND	(–)
*C. dublinensis* subspp. *dublinensis* LMG23823^T^	(+)	(+)	(+)	(+)	(–)	(+)	(–)	(–)	(–)	(–)	(–)	(–)	(–)	(+)	(–)	(–)	(+)
*C. malonaticus* LMG23826^T^	(+)	(+)	(+)	(–)	(–)	(+)	(+)	(–)	(–)	(–)	(–)	(–)	(+)	(–)	(–)	(+)	(+)
*C. turicensis* LMG23827^T^	(+)	(+)	(+)	(–)	(–)	(+)	(+)	(–)	(–)	(–)	(–)	(–)	(+)	(–)	(+)	(+)	(+)
*C. muytjensii* 51329^T^	(–)	(–)	(–)	(–)	(–)	(–)	(–)	(–)	(–)	(–)	(–)	(–)	(–)	(–)	(–)	(–)	(+)
*C. sakazakii* BAA-894	(+)	(+)	(+)	(–)	(+)	(–)	(–)	(+)	(+)	(+)	(+)	(+)	(–)	(–)	(+)	(–)	(+)

Interestingly, only *C. sakazakii* strains GK1025 and Comp59 possessed both the pESA2- and pCTU3-like plasmids; all other strains were PCR-negative for the replicon targets for these two plasmids.

### Phylogenetic analysis of malonate positive Csak ST64 strains

It is thought that microbes engage in complex communication feedback systems with their hosts and environments, although many of the specific mechanisms that link these associations remain unresolved [14]. For example, malonate is a compound particularly abundant in plants such as soybean, and malonate utilization is strongly inducible in plant and bacterial pathogens such as *Xanthomonas axonopodis* pv. *glycines*, the pathogen responsible for bacterial pustule disease [14]. The prevailing hypothesis proposed by Schmid et al. [45] and Joseph et al. [9] is that *Cronobacter* arose during the Paleogene geologic period (between 65 and 23 million years ago, mya) of the Cenozoic era when early modern plants appeared (Paleocene epoch, ~ 65 mya) and the grassland plants (Eocene epoch, ~ 56 mya) continued to evolve.

**Fig. 1:**
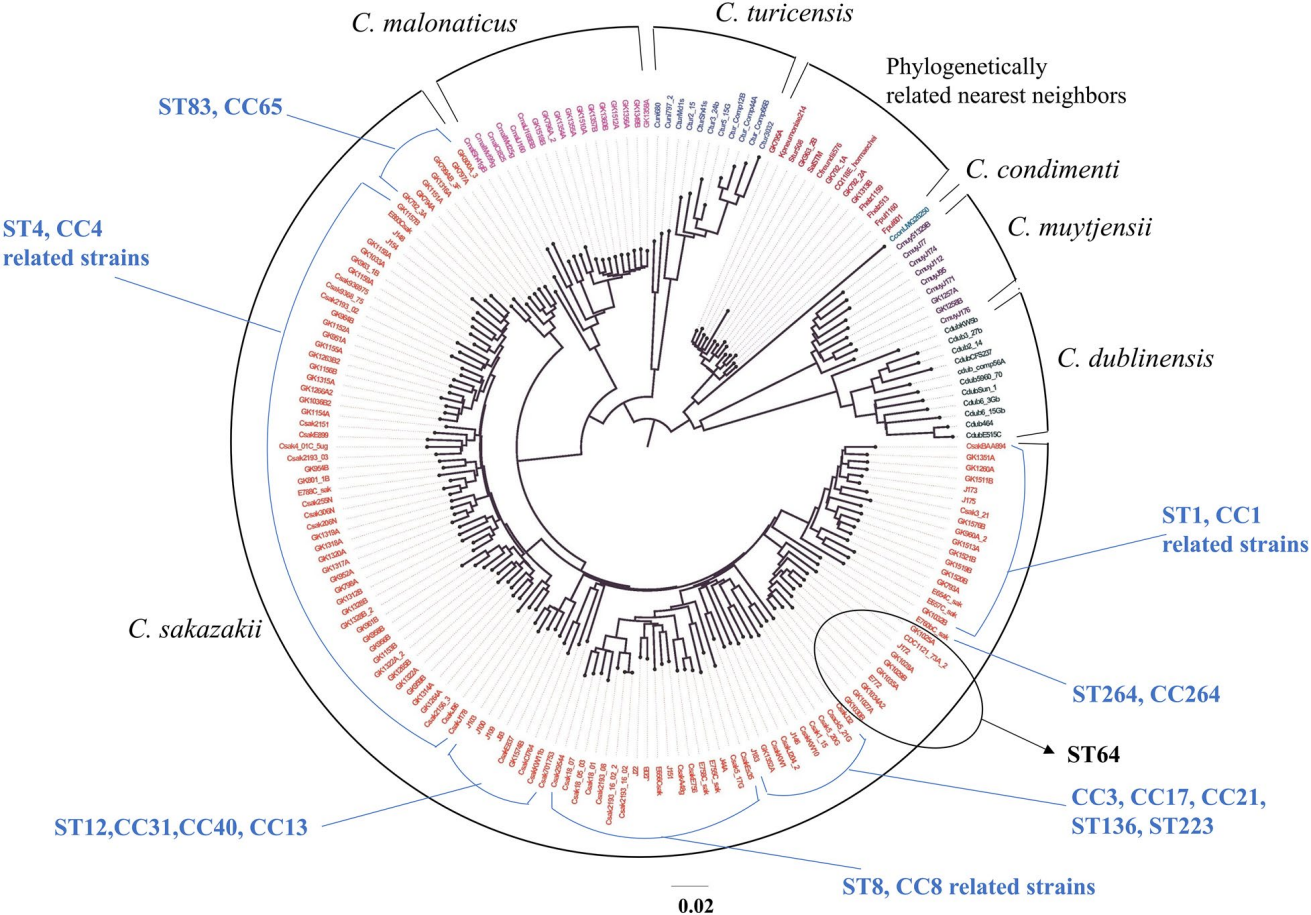
Neighbor net (SplitsTree4) analysis of 188 *Cronobacter* and phylogenetically-related strains which was generated from the microarray-based gene differences that was described by Tall et al. [15]. The phylogenetic tree illustrates that the *Cronobacter* microarray could clearly separate the seven species of *Cronobacter*, with each species forming their own distinct cluster. The ST64 strains cluster together in a single cluster. The scale bar represents a 0.02 base substitution per site

**Fig. 2:**
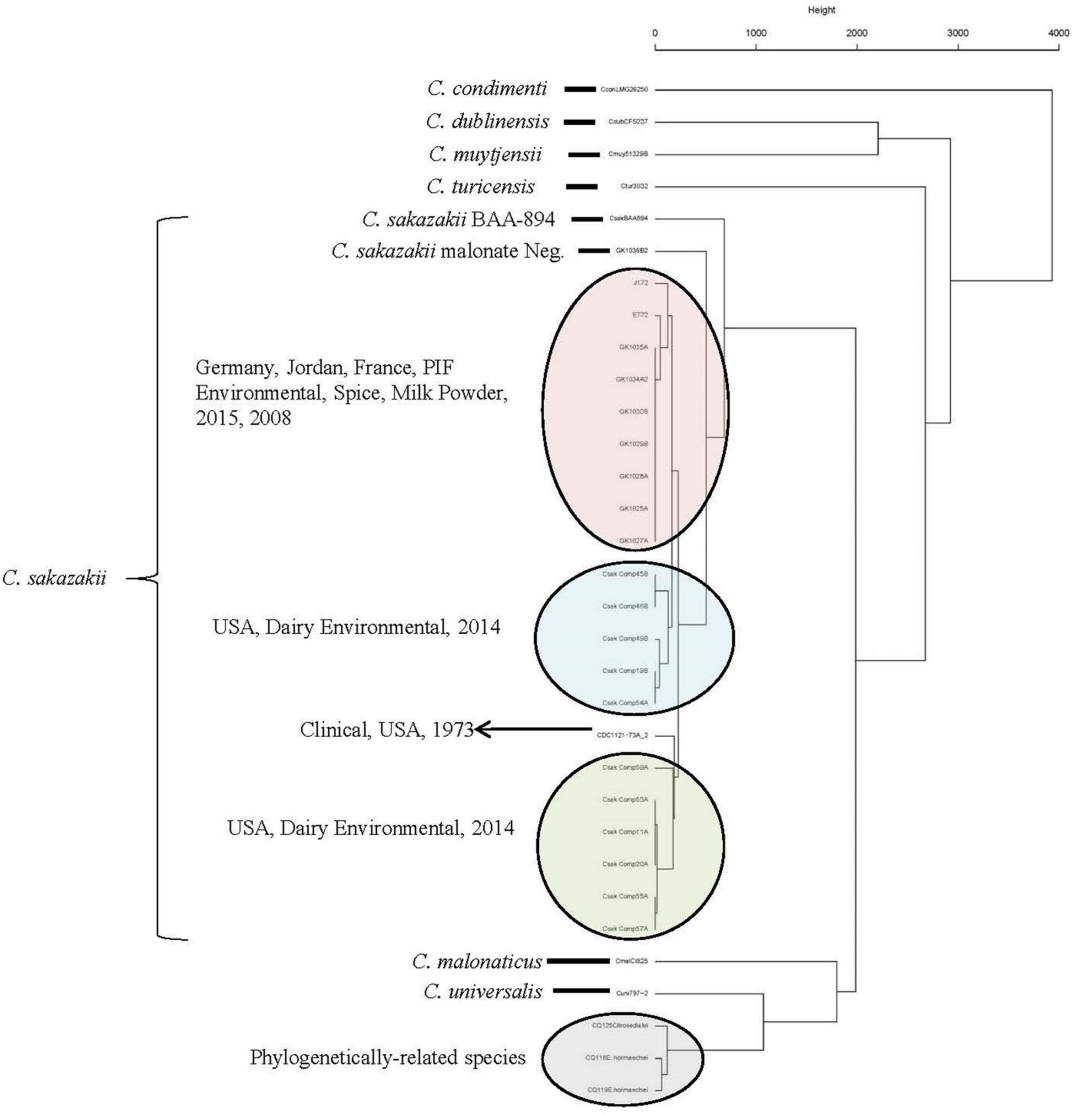
Hierarchical clustering of 21 malonate-positive *C. sakazakii* and phylogenetically-related strains which were generated from the gene difference matrix of the 19,287 independent genes representing 15 *Cronobacter* genomes and 18 plasmids and 2371 virulence factor genes of phylogenetically-related Gram-negative bacteria captured on the pan genomic microarray as described by Tall et al. [15]. The scale bar represents a 0.01 base substitution per site

Results from microarray analysis, shown in Fig. 1 and Additional file 1: Table S1 and Additional file 2: Table S2, (Pearson’s coefficient, gene differences), and Table 3 (list of strains used for MA), revealed that the *C. sakazakii* ST64 strains grouped together within the *C. sakazakii* species cluster and these results support previous microarray findings reported by Tall et al. [15, 41], Yan et al. [13]. and Chase et al. [35] and those findings reported here for the *rpoβ* and *cgcA*-species specific and plasmidotyping PCR and MLST results mentioned earlier. Combinatorial microarray and MLST analyses of the *C. sakazakii* ST64 strains showed that these strains uniquely clustered together (Fig. 1). These results also corroborate a whole genome SNP based tree which was auto generated at the NCBI *C. sakazakii* Genome page and reproduced in Additional file 3: Figure S1 (https://www.ncbi.nlm.nih.gov/genome/tree/1170). Phylogenetic analyses using the whole genome SNP-based tree (Additional file 3: Figure S1) and the pan-genome microarray (Figs. 1, 2) point to at least three major genomic backbone groups of *C. sakazakii* in which sequence types containing clinical strains separated into 2–4 clusters (e.g., ST1, ST4, ST8, and ST83 for example). Malonate positive ST64 *C. sakazakii* strains from this study appear in these trees as a distinct cluster. In addition, the *C. sakazakii* strain Cro2819A (NCBI accession#: NZMBSC00000000 [32] and Table 1) also clustered with the other ST64 *C. sakazakii* strains as developed by the NCBI *C. sakazakii* genome tree.

**Table 3:** *Cronobacter* strains studied using the FDA *Cronobacter* custom designed DNA microarray

Strain name	Species	Source	Serotype	MLST
LMG26520^T^	*C. condimenti*	Food, spiced sausage, Slovakia	ND	98
KW5	*C. dublinensis*	Food, black rice, Republic of Korea	Cdub O:1	ND
3_27	*C. dublinensis*	Food, nuts, Republic of Korea	Cdub O:1	ND
2_14	*C. dublinensis*	Food, nuts, Republic of Korea	Cdub O:1	ND
CFS237	*C. dublinensis*	Environmental, milk production facility, Ireland	Cdub O:1	106
Comp56	*C. dublinensis*	Environmental, compliance activity, USA	Cdub O:1	ND
5960_70	*C. dublinensis*	Clinical, blood	ND	5
SUN_1	*C. dublinensis*	Food, taro, Republic of Korea	ND	ND
6_3G	*C. dublinensis*	Food, nuts, Republic of Korea	Cdub O:1	ND
6_15G	*C. dublinensis*	Food, nuts, Republic of Korea	Cdub O:2	ND
464	*C. dublinensis*	Environmental, milk production facility	ND	79
E515C	*C. dublinensis*	Environmental, water fountain basin	Cdub O:2	80
J160	*C. malonaticus*	Environmental, vacuum dust, Jordan	Csak O:2	ND
J168B	*C. malonaticus*	Environmental, vacuum dust, Jordan	Csak O:2	ND
GK1349	*C. malonaticus*	PIF Facility; Hanover, Germany	Csak O:5	ND
GK1354	*C. malonaticus*	PIF Facility; Hanover, Germany	Csak O:5	ND
GK1355	*C. malonaticus*	PIF Facility; Hanover, Germany	Csak O:5	ND
GK1356	*C. malonaticus*	PIF Facility; Hanover, Germany	Csak O:5	ND
GK1357	*C. malonaticus*	PIF Facility; Hanover, Germany	Csak O:5	ND
GK1358	*C. malonaticus*	PIF Facility; Hanover, Germany	Csak O:5	ND
GK1360	*C. malonaticus*	PIF Facility; Hanover, Germany	Csak O:5	ND
GK1510	*C. malonaticus*	PIF Facility; Hanover, Germany	Csak O:5	ND
GK1512	*C. malonaticus*	PIF Facility; Hanover, Germany	Csak O:5	ND
GK1518	*C. malonaticus*	PIF Facility; Hanover, Germany	Csak O:5	ND
GK796A_2	*C. malonaticus*	PIF Facility; Hanover, Germany	Csak O:5	ND
Sh41g	*C. malonaticus*	Fly, *Sarcophaga haemorrhoidalis*, gut, USA	ND	ND
Md99g	*C. malonaticus*	Fly, *Musca domestica*, gut, USA	Cmal O:1	60
CI825	*C. malonaticus*	Clinical, breast abcess, USA	Cmal O:2	7
Md25g	*C. malonaticus*	Fly, *Musca domestica*, gut, USA	Cmal O:2	7
51329	*C. muytjensii*	Unknown	Cmuy O:2	81
J112	*C. muytjensii*	Food, liquorice, Jordan	ND	ND
GK1257	*C. muytjensii*	PIF Facility; Hanover, Germany	ND	ND
J171	*C. muytjensii*	Food, spices (Fennel), Jordan	ND	ND
GK1258	*C. muytjensii*	PIF Facility; Hanover, Germany	Csak O:1	ND
J174	*C. muytjensii*	Food, spices (Anise), Jordan	Cmuy O:1	ND
J176	*C. muytjensii*	Food, spices (Thyme), Jordan	ND	ND
J77	*C. muytjensii*	Food, spices (Anise), Jordan	Cmuy O:2	ND
J95	*C. muytjensii*	Food, spices (Anise), Jordan	ND	ND
J100	*C. sakazakii*	Food, semolina, Jordan	Csak O:2	ND
J103	*C. sakazakii*	Food, spices, Jordan	Csak O:2	ND
J109	*C. sakazakii*	Food, grapes, Jordan	Csak O:2	ND
J146	*C. sakazakii*	Food, liquorice, Jordan	Csak O:2	ND
GK1025	*C. sakazakii*	PIF Facility; Hanover, Germany	Csak O:2	64
GK1027	*C. sakazakii*	PIF Facility; Hanover, Germany	Csak O:2	64
J148	*C. sakazakii*	Food, spices, Jordan	Csak O:2	4
BAA894	*C. sakazakii*	PIF, USA	Csak O:1	1
GK1028	*C. sakazakii*	PIF Facility; Hanover, Germany	Csak O:2	64
GK1030	*C. sakazakii*	PIF Facility; Hanover, Germany	Csak O:2	64
GK1032	*C. sakazakii*	PIF Facility; Hanover, Germany	Csak O:1	ND
J151	*C. sakazakii*	Food, spices (Fennel), Jordan	Csak O:1	ND
J154	*C. sakazakii*	Food, spices, Jordan	Csak O:3	4
3_21	*C. sakazakii*	Food, nuts, Republic of Korea	Csak O:1	ND
GK1033	*C. sakazakii*	PIF Facility; Hanover, Germany	Csak O:2	ND
GK1034	*C. sakazakii*	PIF Facility; Hanover, Germany	Csak O:2	64
GK1035	*C. sakazakii*	PIF Facility; Hanover, Germany	Csak O:2	64
GK1036	*C. sakazakii*	PIF Facility; Hanover, Germany	Csak O:2	ND
GK1151	*C. sakazakii*	PIF Facility; Hanover, Germany	Csak O:7	ND
GK1152	*C. sakazakii*	PIF Facility; Hanover, Germany	Csak O:2	ND
GK1153	*C. sakazakii*	PIF Facility; Hanover, Germany	Csak O:2	ND
E654	*C. sakazakii*	Clinical, Ireland	Csak O:1	1
E657	*C. sakazakii*	Clinical, Ireland	Csak O:1	1
GK1154	*C. sakazakii*	PIF Facility; Hanover, Germany	Csak O:2	ND
E760	*C. sakazakii*	Clinical, Ireland	Csak O:2	264
GK1155	*C. sakazakii*	PIF Facility; Hanover, Germany	Csak O:2	ND
CDC1121_73	*C. sakazakii*	Clinical, bronchial wash, USA	Csak O:2	64
GK1156	*C. sakazakii*	PIF Facility, Hanover, Germany	Cmal O:2	ND
GK1157	*C. sakazakii*	PIF Facility; Hanover, Germany	Csak o:2	4
E772	*C. sakazakii*	Food, milk powder, France	Csak O:2	64
GK1158	*C. sakazakii*	PIF Facility; Hanover, Germany	Csak O:2	4
GK1159	*C. sakazakii*	PIF Facility; Hanover, Germany	Csak O:2	4
5_21G	*C. sakazakii*	Food, nuts, Republic of Korea	Csak O:2	ND
5_20G	*C. sakazakii*	Food, nuts, Republic of Korea	Csak O:2	ND
1_15	*C. sakazakii*	Food, nuts, Republic of Korea	Csak O:2	ND
KW10	*C. sakazakii*	Food, powdered pine needles, Republic of Korea	Csak O:2	ND
J172	*C. sakazakii*	Food, spices, Jordan	Csak O:2	ND
Es35	*C. sakazakii*	Clinical, Israel	Csak O:1	8
5_17G	*C. sakazakii*	Food, nuts, Republic of Korea	Csak O:1	ND
J173	*C. sakazakii*	Food, spices, Jordan	Csak O:1	ND
E755	*C. sakazakii*	Clinical, Ireland	Csak O:4	8
E758	*C. sakazakii*	Clinical, Ireland	Csak O:4	8
E756	*C. sakazakii*	Clinical, Ireland	Csak O:4	8
A48g	*C. sakazakii*	Fly, *Anthimylidae*, gut, USA	Csak O:6	221
E656	*C. sakazakii*	Clinical, Ireland	Csak O:1	8
J175	*C. sakazakii*	Food, spices, Jordan	Csak O:1	ND
2193_16_02	*C. sakazakii*	Food, nursery water, USA	Csak O:1	8
2193_16_02_2	*C. sakazakii*	Food, nursery water, USA	Csak O:1	8
2193_08	*C. sakazakii*	Food, nursery water, USA	Csak O:1	8
18_01	*C. sakazakii*	Patient, stool sample, USA	Csak O:1	4
18_05_03	*C. sakazakii*	Food, opened can of PIF, USA	Csak O:1	8
18_07	*C. sakazakii*	Patient, stool sample, USA	Csak O:1	8
29544^T^	*C. sakazakii*	Clinical (child), throat swab, USA	Csak O:1	8
701753	*C. sakazakii*	Environmental, PIF plant, USA	Csak O:2	31
KW11	*C. sakazakii*	Food, black bean, Republic of Korea	Csak O:4	ND
CI764	*C. sakazakii*	Clinical, Ireland	Csak O:4	12
GK1260	*C. sakazakii*	PIF Facility; Hanover, Germany	Csak O:1	1
E837	*C. sakazakii*	Clinical, Ireland	Csak O:2	ND
J183	*C. sakazakii*	Food, spices, Jordan	Csak O:1	21
J20	*C. sakazakii*	Food, spices, Jordan	Csak O:1	ND
J204_2	*C. sakazakii*	Food, liquorice, Jordan	Csak O:7	223
J22	*C. sakazakii*	Food, spice (Chamomile), Jordan	Csak O:1	ND
J32	*C. sakazakii*	Food, baby food, Jordan	Csak O:3	ND
J44	*C. sakazakii*	Food, spices, Jordan	Csak O:1	ND
2156_3	*C. sakazakii*	Clinical, blood, USA	Csak O:3	4
GK1263B2	*C. sakazakii*	PIF Facility; Hanover, Germany	Csak O:2	4
GK1264	*C. sakazakii*	PIF Facility; Hanover, Germany	Csak O:2	4
GK1265	*C. sakazakii*	PIF Facility; Hanover, Germany	Csak O:2	ND
GK1266A2	*C. sakazakii*	PIF Facility; Hanover, Germany	Csak O:2	ND
GK1312	*C. sakazakii*	PIF Facility; Hanover, Germany	Csak O:2	ND
GK1314	*C. sakazakii*	PIF Facility; Hanover, Germany	Csak O:2	ND
GK1315	*C. sakazakii*	PIF Facility; Hanover, Germany	Csak O:2	ND
GK1316	*C. sakazakii*	PIF Facility; Hanover, Germany	Csak O:7	ND
GK1317	*C. sakazakii*	PIF Facility; Hanover, Germany	Csak O:2	ND
GK1318	*C. sakazakii*	PIF Facility; Hanover, Germany	Csak O:2	ND
GK1319	*C. sakazakii*	PIF Facility; Hanover, Germany	Csak O:2	ND
GK1320	*C. sakazakii*	PIF Facility; Hanover, Germany	Csak O:2	ND
GK1322	*C. sakazakii*	PIF Facility; Hanover, Germany	Csak O:2	ND
GK1322A_2	*C. sakazakii*	PIF Facility; Hanover, Germany	ND	ND
GK1328B_2	*C. sakazakii*	PIF Facility; Hanover, Germany	Csak O:2	ND
GK1351	*C. sakazakii*	PIF Facility; Hanover, Germany	Csak O:1	ND
206 N	*C. sakazakii*	Clinical, Ireland	Csak O:2	4
306 N	*C. sakazakii*	Clinical, Ireland	Csak O:2	4
255 N	*C. sakazakii*	Clinical, Ireland	Csak O:2	4
E788	*C. sakazakii*	Clinical, Ireland	Csak O:2	4
GK1352	*C. sakazakii*	PIF Facility; Hanover, Germany	Csak O:1	ND
2193_03	*C. sakazakii*	Clinical, CSF, USA	Csak O:2	4
4_01C_5ug	*C. sakazakii*	Food, PIF	Csak O:2	218
E899	*C. sakazakii*	Clinical, USA	Csak O:2	4
2151	*C. sakazakii*	Clinical, USA	Csak O:2	4
GK1511	*C. sakazakii*	PIF Facility; Hanover, Germany	Csak O:1	ND
GK1513	*C. sakazakii*	PIF Facility; Hanover, Germany	Csak O:1	ND
2193_02	*C. sakazakii*	Clinical, sputum, USA	Csak O:2	4
9368_75	*C. sakazakii*	Unknown	Csak O:2	4
9369_75	*C. sakazakii*	Unknown	Csak O:4	4
GK1519	*C. sakazakii*	PIF Facility; Hanover, Germany	csak O:1	ND
GK1520	*C. sakazakii*	PIF Facility; Hanover, Germany	Csak O:1	ND
GK1521	*C. sakazakii*	PIF Facility; Hanover, Germany	Csak O:1	ND
GK1574	*C. sakazakii*	PIF Facility; Hanover, Germany	Csak O:4	ND
J93	*C. sakazakii*	Food, spices, Jordan	Csak O:2	ND
E893	*C. sakazakii*	Clinical, Ireland	Csak O:2	4
GK1576	*C. sakazakii*	PIF Facility; Hanover, Germany	Csak O:1	ND
GK792_3	*C. sakazakii*	PIF Facility; Hanover, Germany	Csak O:7	83
GK793	*C. sakazakii*	PIF Facility; Hanover, Germany	Csak O:1	1
GK794	*C. sakazakii*	PIF Facility; Hanover, Germany	Csak O:7	83
J96	*C. sakazakii*	Food, spices (Fennel), Jordan	Csak O:3	ND
GK797	*C. sakazakii*	PIF Facility; Hanover, Germany	Csak O:7	ND
GK798	*C. sakazakii*	PIF Facility; Hanover, Germany	Csak O:2	4
GK799AB_3F	*C. sakazakii*	PIF Facility; Hanover, Germany	Csak O:7	83
GK800	*C. sakazakii*	PIF Facility; Hanover, Germany	Csak O:7	83
GK801	*C. sakazakii*	PIF Facility; Hanover, Germany	Csak O:2	4
GK951	*C. sakazakii*	PIF Facility; Hanover, Germany	Csak O:2	4
GK952	*C. sakazakii*	PIF Facility; Hanover, Germany	Csak O:2	4
GK954	*C. sakazakii*	PIF Facility; Hanover, Germany	Csak O:2	4
GK956	*C. sakazakii*	PIF Facility; Hanover, Germany	Csak O:2	4
GK958	*C. sakazakii*	PIF Facility; Hanover, Germany	Csak O:2	4
GK959	*C. sakazakii*	PIF Facility; Hanover, Germany	Csak O:2	4
GK960	*C. sakazakii*	PIF Facility; Hanover, Germany	Csak O:1	ND
GK961	*C. sakazakii*	PIF Facility; Hanover, Germany	Csak O:2	4
GK963_1	*C. sakazakii*	PIF Facility; Hanover, Germany	Csak O:2	ND
GK964	*C. sakazakii*	PIF Facility; Hanover, Germany	Csak O:2	4
680	*C. turicensis*	Food, unspecified	ND	48
797	*C. universalis*	Environmental, water, UK	Cuni O:1	54
Md1 s	*C. turicensis*	Fly, *Musca domestica*, surface, USA	ND	7
2_15	*C. turicensis*	Food, nuts, Republic of Korea	ND	ND
Sh41 s	*C. turicensis*	Fly, *Sarcophaga haemorrhoidalis*, gut, USA	ND	ND
3_24	*C. turicensis*	Food, nuts, Republic of Korea	Csak O:5	ND
5_15G	*C. turicensis*	Food, nuts, Republic of Korea	Csak O:5	ND
Comp12	*C. turicensis*	Environmental, compliance activity, USA	ND	24
Comp44	*C. turicensis*	Environmental, compliance activity, USA	ND	24
Comp66	*C. turicensis*	Environmental, compliance activity, USA	Ctur O:1	ND
3032	*C. turicensis*	Clinical (neonate), blood, Switz.	Ctur O:1	19
576	*C. freundii*	Unknown	ND	ND
CQ118	*E. hormaechei*	PIF Facility; Dublin Ireland	ND	ND
1159/04	*F. helveticus*	LMG 23733, fruit punch powder, Switz.	ND	ND
513/05^T^	*F. helveticus*	LMG 23732T, fruit punch powder, Switz.	ND	ND
1160/04	*F. pulveris*	LMG 24058, fruit punch powder, Switz.	ND	ND
601/05^T^	*F. pulveris*	LMG 24057T, fruit punch powder, Switz.	ND	ND
508/05^T^	*S. turicensis*	LMG 23730T, fruit punch powder, Switz.	ND	ND
214	*K. pneumoniae*	Unknown	ND	ND
SalSTM	*Salmonella typhimurium*	Unknown	ND	ND
GK1313	Non-*Cronobacter*	PIF Facility; Hanover, Germany	ND	ND
GK792_1	Non-*Cronobacter*	PIF Facility; Hanover, Germany	ND	ND
GK792_2	Non-*Cronobacter*	PIF Facility; Hanover, Germany	ND	ND
GK795	Non-*Cronobacter*	PIF Facility; Hanover, Germany	ND	ND
GK963_2	Non-*Cronobacter*	PIF Facility; Hanover, Germany	ND	ND

**Fig. 3:**
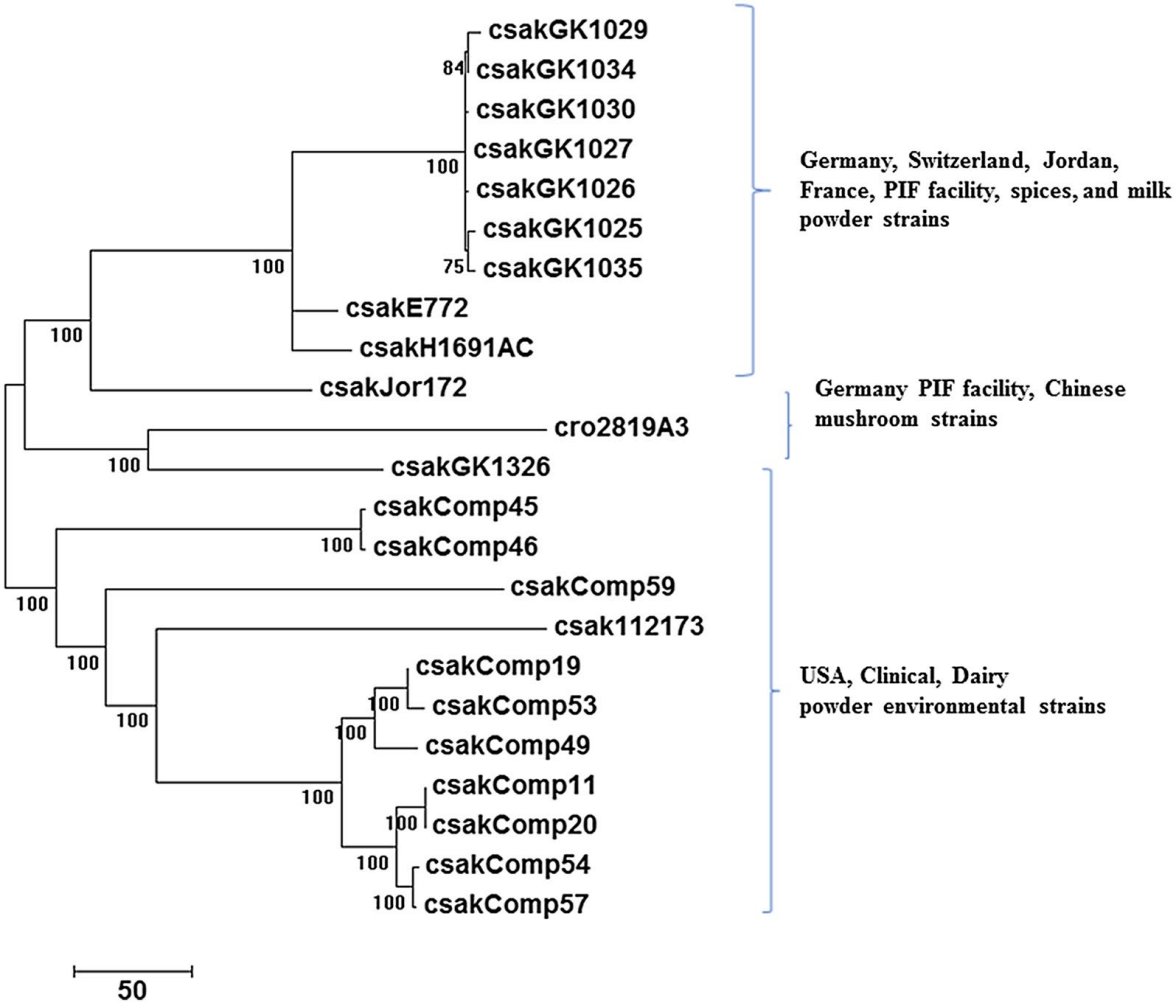
kSNP3 whole genome SNP analysis. Using the kSNP3 [33] SNP-finding tool, 1.6 million positions with SNP information across 23 malonate-positive *C. sakazakii* genomes were identified. The average size of *C. sakazakii* genome used in this analysis was approximately 4.54 MB and approximately 220,000 SNP positions across these genomes were used in the analysis. The phylogenetic tree was generated using the SNP-matrix algorithm provided by the kSNP3 tool. The numbers in the nodes denote bootstrap values. Mega 7.0 without gaps was used to align the sequences and develop the phylogenetic tree. The ST64 *C. sakazakii* strains grouped into two major clusters: the first comprised strains from the German PIF manufacturing facility; and the second cluster was comprised of strains which came from the USA diary powder manufacturing facilities. Several *C. sakazakii* ST64 strains such as E772, H1691AC, and Jor172 which came from a milk powder sample from France; a Swiss PIF manufacturing facility sample; and a Jordanian spice sample share greater phylogeny with that of the German PIF strains, respectively. Additionally, three USA diary powder manufacturing facility strains and the clinical strain from the USA were also more related to the European and Middle East strains than the second cluster of USA diary powder manufacturing facilities strains. Bar marker represents 0.00020 nucleotide differences per 10,000 positions

We characterized these genomes further by datamining microarray datasets and by whole genome SNP detection approaches. Total gene differences observed among the USA ST64 strains included between 0 and 216 genes (Additional file 1: Table S1, Additional file 2: Table S2). There were no differences in total genes among the German PIF manufacturing facility strains and only 45–125 genes between the German strains and the French milk powder and Jordan spice strains. This suggests that the strains obtained during the German PIF manufacturing facility surveillance study were phylogenetically-related to one another. To understand the strain-level genomic variations among the strains of ST64 group from this study, we carried out whole genome SNP analysis using our de novo assemblies reported in this study, and those reported earlier by Chase et al. [35] and the genome of *C. sakazakii* strain Cro2819A3 (Table 1). As indicated in Fig. 3, the whole genome analysis using the kSNP3 algorithm showed a similar pattern of phylogenetic relatedness among the strains where the German PIF strains (Csak GK-series) clustered together (some indistinguishable among them) with the other European and Jordanian strains along with the Chinese ST64 *C. sakazakii* strain Cro2819A3. Although these strains were more phylogenetically diverse among themselves; they were distinct from the USA dairy powder and clinical strains. This pattern of clustering based on varying degrees of phylogenetic relatedness was supported by the pan-genomic microarray analysis (Fig. 2). The low degree of genomic diversity among ST64 strains observed in Fig. 2 can be explained as an inherent feature resulting from random acquisition of “mobilome” genes representative of the dispensable genome such as plasmids and phage sequences as proposed by Joseph et al. [9] and Zheng et al. [27]. The pan-genomic microarray [15] contains probeset sequences that cover both chromosomal and mobilome genomes, representative of *Cronobacter* genus. In contrast, analysis of whole genome SNPs, based on genome assemblies, is driven almost entirely by chromosomal sequences. A similar genus-wide comparison using 2500+ chromosomal core genes clustered the ST64 strains into a single group (data not shown) as predicted and indicated a generally conserved backbone of these strains with minimal differences when compared to other *C. sakazakii* strains, and that the mobilome of this diverse group contributes to the observed differences. It should also be noted that of the 122 *C. sakazakii* strains (representing multiple STs) that were evaluated by microarray analysis, as described in this study, only the ST64 *C. sakazakii* strains were found to possess the malonate operon. Additionally, we performed a BLAST analysis on several hundred *C. sakazakii* strains in our genomic collection (data not shown), and to date; only ST64 *C. sakazakii* strains have been found to be malonate positive both phenotypically and genotypically. It is evident from these analyses that the ST64 *C. sakazakii* strains: (a) form a tight phylogenetic cluster, and (b) share a genomic backbone closer to other *C. sakazakii* than that with other *Cronobacter* species while exhibiting genomic differences among themselves.

### Genomic characterization of the malonate utilization operon in ST64 *C. sakazakii* strains

**Fig. 4:**
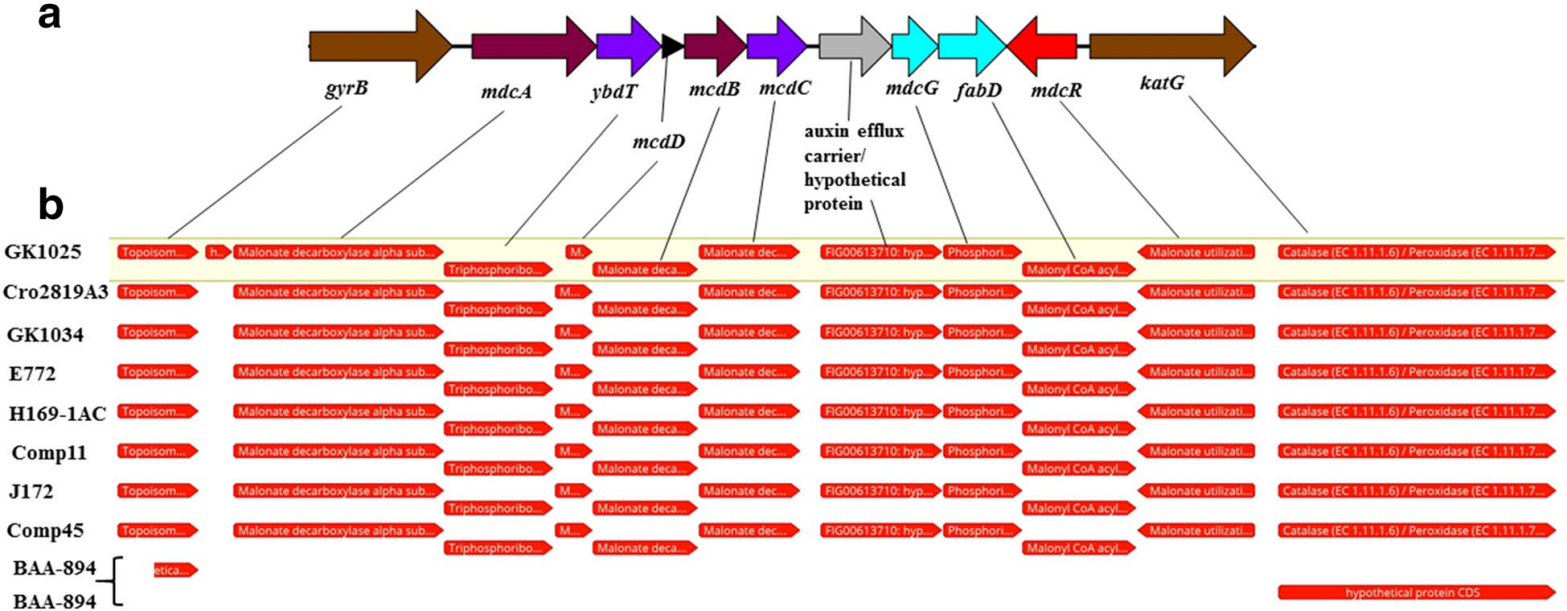
**a** Malonate utilization operon annotated from ST64 *C. sakazakii* strain GK1025B. The schematic representation of the nine gene malonate utilization operon. **b** Comparison of the malonate utilization operon from different strains of ST64 isolates: The reference strain GK1025B was previous reported in Chase et al. [18] and Cro2819A3 was reported by Zheng et al. [27]. Only the partial sequence of topoisomerase was included for illustration. BAA-894 fragments in the last two tracks are contiguous in its genome separated by a 325 bp intergenic region; the operon is inserted in this spacer region in other species. Comparative genomic analysis of the malonate operon allele sequences from eight ST64 malonate-positive *C. sakazakii* strains compared to that of malonate-negative *C. sakazakii* strain BAA-894. A similar operon structure was found for all 23 strains. The ST64 *C. sakazakii* malonate utilization operon is flanked upstream by *gyrB* which encodes a topoisomerase IV subunit B gene (EC 5.99.1), and downstream by *katG*, encoding a catalase/peroxidase gene (EC 1.11.1.6; EC 1.11.1.7). The malonate utilization gene cluster-flanking regions were found to be conserved among all *Cronobacter* species even malonate-negative *C. sakazakii* strains; however, instead of the 7.7 kbp malonate utilization gene cluster, there is a 323–325 bp nucleotide region. The operon contains genes encoding enzymes and proteins involved in the decarboxylation of malonate and includes a malonate utilization transcriptional regulator *mdcR*, malonate decarboxylase consists of the oligomerization of alpha, delta, beta, and gamma protein subunits and is encoded by four genes, *mdcADBC.* Finally, this gene cluster also contains genes encoding for a 2-(5′-triphosphoribosyl)-3′-dephosphocoenzyme-A synthase (*ybdT*), malonyl CoA acyl carrier protein transacylase (*fabD*) and a phosphoribosyl-dephospho-CoA transferase (*mdcG*) which are thought to stabilize the coenzyme-A complex, and may allow the proper conformation of malonate decarboxylase to be maintained in a high substrate affinity configuration

RAST/SEED analysis of the malonate utilization operon of *C. sakazakii* ST64 strain 1121-73 showed that it was ~ 7.7 kbp in size and revealed a cluster of nine genes. A summary of these genes is shown in Additional file 4: Table S3. A physical map of the malonate utilization operon is shown in Fig. 4a. The operon is flanked upstream by *gyrB* which encodes a topoisomerase IV subunit B gene (EC 5.99.1), and downstream by *katG*, encoding a catalase/peroxidase gene (EC 1.11.1.6; EC1.11.1.7). When the operon sequences from selected strains representing this heterogenous group as shown in Fig. 3 were compared with *C. sakazakii* strain GK1025B, it was clear that the ST64 strains retained this conserved operon structure (Fig. 4b). Also, the malonate utilization gene cluster-flanking regions were found to be conserved among all *Cronobacter* species, even malonate-negative *C. sakazakii* strains like *C. sakazak*ii strain BAA-894 (Fig. 4b bottom two tracks); however, the 7.7 kb malonate utilization gene cluster in these strains is instead replaced with a 323–325 bp nucleotide region, and is represented by the nucleotide region (bp position 1723016 to 1723340 in BAA-894 NCBI GenBank accession no: CP000783). This study identified for the first time a functional malonate utilization operon regulated by *mdcR* in *C. sakazakii* ST64 strains, which is highly similar to the operon described by Koo et al. for *Acinetobacter calcoaceticus* [46]. However, it seems to be different than that possessed by the *matR*-regulated malonate *matABC* operon found in *Rhizobium* spp. in both size (7.7 kbp versus 7.0 kbp) and operon structure (9 genes versus 4 genes). MatR plays a dual role in the transcription of *matR* and *matABC* with malonate as a positive effector [47, 48]. Also, *matA* encodes malonyl-CoA decarboxylase, whereas *matB* encodes malonyl-CoA synthetase. The MatC protein appears to be an integral membrane protein that can function as a malonate transporter [46].

**Fig. 5:**
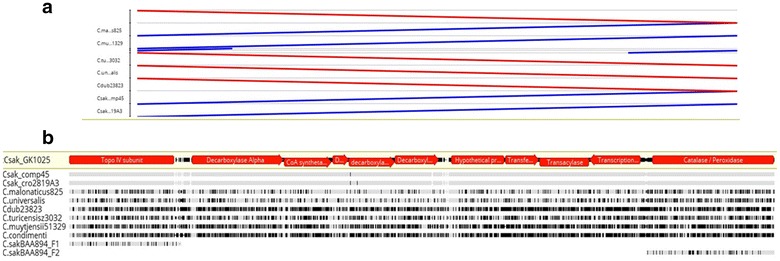
**a** LASTZ pairwise alignment analysis of the *Cronobacter* malonate utilization operon. 11.5 kb of the operon from all the listed strains were subjected to LASTZ algorithm implemented in Geneious suite. The lines indicate conserved blocks in the pairwise alignment of each operon sequence with *C. sakazakii* strain GK1025 sequences. The length of the lines indicates the extent of conservation ignoring coding bias. **b** Multiple alignment analysis of the *Cronobacter* malonate utilization operon. 11.5 kb of the operon from all the listed strains were aligned with the reference *C. sakazakii* strain GK1025 sequences. The gray horizontal bar indicate sequence similarity and black vertical bar point to possible alleles. Significant allelic differences result in clusters of black patches in some tracks. Flanking alleles from *C. sakazakii* strain BAA-894 F1 (*gyrB*) and F2 (*katG*) are contiguous in the genome but are separated by a 325 bp intergenic spacer. In other strains used in this illustration, the operon is seen to have inserted in this spacer region

Next, we analyzed the genomes of the ST64 *C. sakazakii* strains using whole genome sequences to understand the evolutionary relationship among malonate utilization operons found associated with other species of *Cronobacter*. The pan-genomic-based *Cronobacter* microarray described by Tall et al. [15] contains 50 probe sets representing orthologous alleles from *C. dublinensis* subsp. *dublinensis* strain LMG23823^T^, *C. muytjensii* strain 51329^T^, *C. malonaticus* strain LMG23826^T^, *C. universalis* strain NCTC9529^T^, and *C. turicensis* strain LMG23827^T^. Additional file 5: Table S4 shows microarray analysis of ST64 *C. sakazakii* strains using these probe sets alone. The results predict a significant degree of sequence homology (as inferred from interpreting the presence/absence calls) among these orthologous alleles. The malonate utilization operon containing contigs were identified in all the genomes of the ST64 strains from Table 1 by querying them with the annotated operon from GK1025B. BLAST analysis, reference mapping with Geneious, and RAST table comparisons yielded a fully annotated operon in the 24 ST64 *C. sakazakii* strains, and six other *Cronobacter* species, excluding *C. sakazakii* strain BAA-894. Based on our observations (from Figs. 2, 3) about the genomic differences among the *Cronobacter* species and related data, we analyzed the sequence similarity of the nucleotide sequences encoding the operon in the six other *Cronobacter* species using the LASTZ algorithm as implemented in Geneious. This presents both the LASTZ pairwise alignment blocks on the top and multiple alignments annotating the alleles in the bottom panel. The LASTZ alignment (Fig. 5a) ignores coding sequence bias [31] to create optimal conserved blocks. The reference mapping tool highlights allelic differences in each strain when compared against the reference ST64 *C. sakazakii* strain GK1025 genome. The results point to a genomic region with conserved blocks of sequences (Fig. 5a) in spite of extensive nucleotide differences (Fig. 5b) seen in distant species. It is apparent from this analysis that the malonate utilization genomic region has undergone independent evolution among the various *Cronobacter* species with *C. malonaticus* and *C. universalis* appearing to be more similar to the ST64 *C. sakazakii* malonate operon than that of the other species. This level of genomic difference among the species is consistent with other reports from our group [16, 19, 35] and others [9]. Using BLAST analysis and reference mapping, the gene content of the operon in all *Cronobacter* species appear to be preserved with conserved amino acid sequence (data not shown) across the species. As a use case to illustrate this, we compared the amino acid sequences of malonate decarboxylase (Mdc) from all the species using Clustal Omega. The resulting alignment, shown in Additional file 6: Figure S2, points to a highly conserved protein with only a few changes in the amino acid moieties. This is reflected in minimal number of changes in the amino acid composition between *C. sakazakii* strain GK1025B and *C. condimenti* strain 1330, the two phylogenetically distant organisms seen from this multiple alignment (Additional file 6: Figure S2).

Taken together, these data suggest that the origins of the *C. sakazakii* ST64 malonate operon can be explained in a couple of hypotheses. One hypothesis may be that this operon was acquired after the predicted evolutionary event [16] when *C. sakazakii*, *C. universalis*, *C. turicensis* and *C. malonaticus* evolved from their primordial ancestor during the event that led to them jumping host species e.g., from their plant-associated host to a secondary host. Furthermore, an insect vector such as the common filth fly as suggested by Lehner et al. may have been involved [49]. This possibility alludes to a post-speciation gain-of-function adaptation of the ST64 lineage of *C. sakazakii*. The 325-bases pair region following the *gryB* coding gene in (Figs. 4, 5) the genome of *C. sakazakii* strain BAA-894 is also seen in many *Enterobactereaceae* members lacking the malonate utilization phenotype (GG, personal communication) and offers support of this hypothesis. Under this hypothesis, the operon originating from a hitherto unidentified parent might have been inserted into the ST64 *C. sakazakii* lineage as it adapted to changing host preferences and making it a more recent event. Alternatively, the conservation of this operon across every *Cronobacter* species also points to an older genomic feature inherited from earlier ancestral stock and was retained initially in all *Cronobacter* species. During evolution with the exception of ST64 *C. sakazakii* group, all other lineages of *C. sakazakii* of the now non-plant host-adapted lineages might have lost this operon reflecting a cessation of a need-to-retain malonate utilization phenotype, a loss-of-function evolutionary event. Speculatively, this would render the ST64 clade to be one of the oldest *C. sakazakii* lineages, retaining specific plant-associated features of the primordial ancestor of the genus, but phylogenetically placed with other *C. sakazakii* lineages which have clearly adapted to more modern hosts and environmental niches. A comprehensive molecular evolutionary analysis of a few hundred genomes may shed light on this critical aspect of the evolutionary history of *Cronobacter*.

### Zebrafish embryo infection studies show that *C. sakazakii* ST64 strains are virulent

**Fig. 6:**
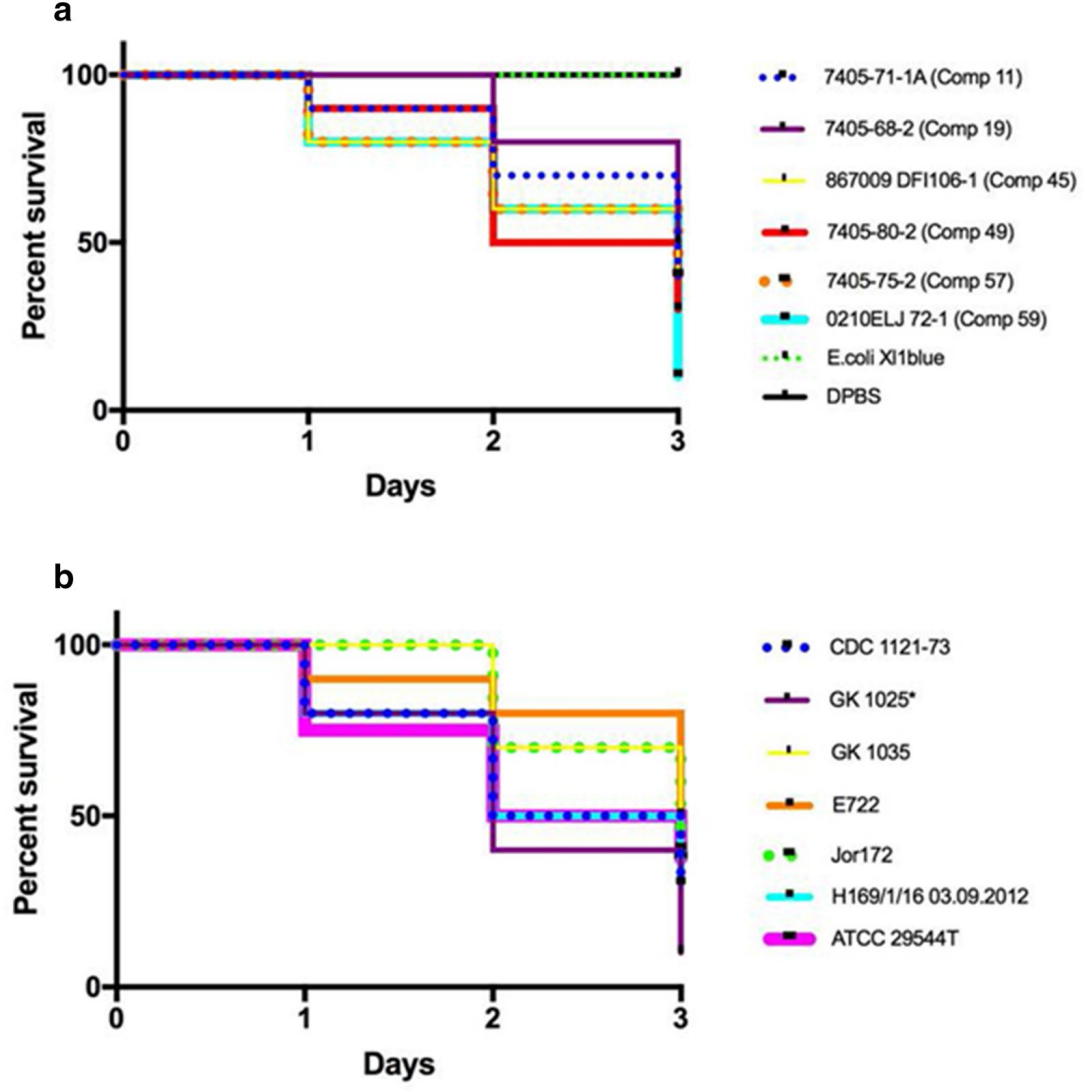
Results of zebrafish embryo infection (time course 0–72 hpi) experiments with 12 malonate-positive *C. sakazakii* ST64 strains, the two clinical strains ATCC29544^T^ and 1121–73 as well as *E. coli* Xl1 blue

By 72 h post injection, all 12 ST 64 strains were lethal to zebrafish embryos; nine of the strains presented at least a 50% mortality rate with two strains (GK1025 and Comp 59) possessing a 90% mortality rate (Fig. 6a, b). This data provide evidence that Csak O:2, ST64, malonate-positive *C. sakazakii* strains are as virulent as other *Cronobacter* species and non malonate-utilizing *C. sakazakii* strains, as reported by Eshwar et al. [8] and Chase et al. [35].

## Conclusions

Until this investigation, the presence of plant and food-associated, malonate-positive *C. sakazakii* strains was under appreciated, possibly leading to misidentification when relying on phenotypic analysis alone. A custom designed pan genome microarray was useful in characterizing the total genome content of 23 Csak O:2, ST64, malonate-positive strains; and showed for the first time that these *C. sakazakii* strains were highly related in total gene content, serotype, and ST. To date, DNA microarray and WGS analyses clearly demarcated only the ST64 group of *C. sakazakii* strains as being genotypically positive for the malonate utilization operon; and phenotypically, these strains were found to utilize malonate. Genus wide, the microarray correctly pointed to other genotypic malonate-positive *Cronobacter* species, namely *C. malonaticus*, *C. turicensis*, *C. universalis*, *C. muytjensii*, *C. condimenti*, and *C. dublinensis* possessing the alleles of this operon. As far as whether or not *C. sakazakii* strains of other STs possess the alleles we have not seen this in any other *C. sakazakii* strains. Other researchers have found *C. sakazakii* ST64 strains which were associated with plant-origin foods, but no mention of malonate utilization has been reported [27]. BLAST analysis of more than 140 genomes (from this study; Ref. [27] and many genomes with known ST represented in Additional file 3: Figure S1) clearly pointed only to the ST64 strains possessing this operon and non-ST64 strains were negative. As an emerging pathogen, the host range of *Cronobacter* in general, *C. sakazakii* in particular, is expanding and future surveillance studies should help answer this important hitherto unanswered question. This study further establishes parallel MA and WGS analyses as powerful platforms for genomics research of *Cronobacter* while generally underscoring the applicability of WGS data to answer both biological and evolutionary questions about the members of this emerging pathogenic genus. The data collected in this study increases the number of publicly available *Cronobacter* genomes, including malonate-positive *C. sakazakii* strains isolated from both powdered infant formula and dairy powder manufacturing environments, dried milk powders, spices, and clinical sources. This study clearly establishes the power of genomic data from *Cronobacter* strains that can be harnessed to develop rapid and inexpensive next generation molecular diagnostics. The data presented also supports the establishment of the Zebrafish embryo infection model and its ability to play a key role in high throughput comparative genomics experiments to help unveil the virulence determinants of *Cronobacter* spp. that contribute to human disease.

## Additional files

**Additional file 1: Table S1.** Pearson’s correlation anlaysis of malonate-positive *C. sakazakii* strains compared to other *Cronobacter* and non *Cronobacter* strains.

**Additional file 2: Table S2.** Gene difference analysis of malonate-positive *C. sakazakii* strains compared to other *Cronobacter* and non *Cronobacter* strains.

**Additional file 3: Figure S1.** This phylogenetic tree was downloaded from the NCBI website (https://www.ncbi.nlm.nih.gov/genome/tree/1170) which is part of the NCBI *C. sakazakii* genome tree report resource provided by NCBI and then ST information for each strain in the tree was obtained from the *Cronobacter* MLST website (http://pubmlst.org/cronobacter/) was manually overlaid onto the tree. The tree/dendrogram in Entrez Genome (https://www.ncbi.nlm.nih.gov/genome/) was developed using pairwise GenBank assembly sequence comparisons as a result of megablast alignments. The pairwise distance is retrieved from the BLAST results by a gpipe script as Symmetric identity (which is converted to the distance between assemblies). Personal communication of NCBI’s phylogenetic analysis was provided by Boris Fedorov, Igor Tolstoy, Tatiana Tatusova, and Richa Agarwala, NIH/NLM/NCBI.

**Additional file 4: Table S3.** Description of malonate utilization operon in ST64 *Cronobacter* strains described in this study.

**Additional file 5: Table S4.** Additional table.

**Additional file 6: Figure S2.** Clustal analysis of *mdcB* protein from ST64 and non-*C. sakazakii* strains. Proteins sequences were retrieved from the annotations found in NCBI or RAST/SEED server originally carried out as part of this study. The sequences were subjected Clustal Omega multiple alignment. Amino acid substitutions in different species are noted in the illustration reflecting the nucleotide diversity discussed elsewhere.

## Abbreviations

MA: microarray analysis
WGS: whole genome sequencing
ST: sequence type
Csak O:2: *C. sakazakii* serotype O:2
PIF: powdered infant formula
USA: United States of America
GR: genome region
*gryB*: topoisomerase IV subunit B gene (EC 5.99.1)
*katG*: catalase/peroxidase gene
*mdcR*: malonate utilization transcriptional regulator
*mdcF*: a malonate transporter gene
*mdcE*: which encodes for a stabilization protein
*mdcADBC*: alpha, delta, beta, and gamma subunit genes of malonate decarboxylase
*ybdT*: 2-(5′-triphosphoribosyl)-3′-dephosphocoenzyme-A synthase
*fabD*: malonyl CoA acyl carrier protein transacylase
*mdcG*: phosphoribosyl-dephospho-CoA transferase
FDA: Food and Drug Administration
*zpx*: zinc metalloprotease
*rpoβ*: β subunit of bacterial RNA polymerase
*cgcA*: diguanylate cyclase-encoding gene
PCR: polymerase chain reaction
MLST: multiple locus sequence typing
