# Supplementary material for: Genomic characterization of malonate positive Cronobacter sakazakii serotype O:2, sequence type 64 strains, isolated from clinical, food, and environment samples
Source: Gut Pathog. 2018 Mar 10;10:11. doi: 10.1186/s13099-018-0238-9 (PMC5845375; doi:10.1186/s13099-018-0238-9)
Supplement: Supplementary file 1 — Additional file 1: Table S1. Pearson’s correlation anlaysis of malonate-positive C. sakazakii strains compared to other Cronobacter and non Cronobacter strains. [file 13099_2018_238_MOESM1_ESM.pdf]

Supplemental Table 1. Pearson's correlation anlaysis of malonate-positive *C. sakazakii* strains compared to other *Cronobacter* and non *Cronobacter* strains*.

Strains	CsakBAA894	Csak Comp54A	Csak Comp19B	Csak Comp11A	Csak Comp20A	Csak Comp53A	Csak Comp45B	Csak Comp46B	Csak Comp49B	Csak Comp57A	Csak Comp59A	E772	GK1025A	GK1027A	GK1028A	GK1029B	GK1030B	GK1034A2	GK1035A	CDC1121-73A_2	J172	Ctur3032	Cuni797-2	CconLMG26250	CdubCFS237	CmalCI825	Cmuy51329B	CQ118E. hormaeche	CQ119E.hormaechei	CQ125Citrosedlakii
CsakBAA894	1.00	0.74	0.74	0.82	0.78	0.81	0.80	0.80	0.80	0.80	0.81	0.81	0.80	0.81	0.82	0.82	0.81	0.79	0.81	0.82	0.82	0.45	0.51	0.28	0.30	0.58	0.30	0.40	0.42	0.40
Csak Comp54A	0.74	1.00	0.97	0.83	0.91	0.85	0.84	0.81	0.84	0.90	0.88	0.85	0.83	0.84	0.82	0.82	0.85	0.85	0.85	0.82	0.85	0.37	0.48	0.21	0.22	0.52	0.27	0.31	0.30	0.27
Csak Comp19B	0.74	0.97	1.00	0.82	0.90	0.85	0.84	0.81	0.83	0.90	0.87	0.84	0.82	0.84	0.82	0.82	0.85	0.85	0.83	0.82	0.84	0.37	0.47	0.22	0.22	0.52	0.27	0.31	0.30	0.27
Csak Comp11A	0.82	0.83	0.82	1.00	0.92	0.96	0.94	0.93	0.92	0.94	0.91	0.91	0.91	0.92	0.93	0.94	0.92	0.90	0.92	0.93	0.92	0.47	0.54	0.31	0.31	0.62	0.31	0.44	0.44	0.44
Csak Comp20A	0.78	0.91	0.90	0.92	1.00	0.94	0.91	0.87	0.86	0.96	0.93	0.89	0.86	0.91	0.89	0.89	0.91	0.91	0.90	0.88	0.90	0.43	0.52	0.26	0.27	0.58	0.29	0.40	0.38	0.37
Csak Comp53A	0.81	0.85	0.85	0.96	0.94	1.00	0.95	0.92	0.89	0.96	0.93	0.92	0.88	0.94	0.93	0.93	0.93	0.93	0.91	0.92	0.92	0.46	0.54	0.30	0.30	0.61	0.30	0.43	0.43	0.42
Csak Comp45B	0.80	0.84	0.84	0.94	0.91	0.95	1.00	0.95	0.89	0.93	0.93	0.91	0.88	0.94	0.93	0.93	0.93	0.93	0.91	0.92	0.93	0.46	0.54	0.31	0.31	0.61	0.31	0.41	0.41	0.39
Csak Comp46B	0.80	0.81	0.81	0.93	0.87	0.92	0.95	1.00	0.92	0.89	0.88	0.90	0.91	0.91	0.92	0.92	0.90	0.88	0.91	0.92	0.91	0.47	0.54	0.32	0.33	0.62	0.32	0.42	0.44	0.43
Csak Comp49B	0.80	0.84	0.83	0.92	0.86	0.89	0.89	0.92	1.00	0.88	0.86	0.89	0.92	0.88	0.90	0.90	0.88	0.85	0.90	0.92	0.90	0.46	0.53	0.30	0.32	0.61	0.32	0.41	0.43	0.42
Csak Comp57A	0.80	0.90	0.90	0.94	0.96	0.96	0.93	0.89	0.88	1.00	0.95	0.92	0.87	0.93	0.91	0.91	0.92	0.93	0.91	0.91	0.92	0.45	0.53	0.27	0.28	0.60	0.30	0.41	0.40	0.38
Csak Comp59A	0.81	0.88	0.87	0.91	0.93	0.93	0.93	0.88	0.86	0.95	1.00	0.92	0.87	0.93	0.91	0.91	0.93	0.93	0.90	0.90	0.92	0.45	0.53	0.28	0.28	0.60	0.30	0.43	0.42	0.39
E772	0.81	0.85	0.84	0.91	0.89	0.92	0.91	0.90	0.89	0.92	0.92	1.00	0.92	0.95	0.95	0.95	0.95	0.94	0.94	0.92	0.93	0.45	0.54	0.29	0.29	0.62	0.31	0.39	0.40	0.39
GK1025A	0.80	0.83	0.82	0.91	0.86	0.88	0.88	0.91	0.92	0.87	0.87	0.92	1.00	0.92	0.94	0.95	0.93	0.90	0.95	0.90	0.91	0.45	0.53	0.29	0.31	0.62	0.32	0.40	0.42	0.40
GK1027A	0.81	0.84	0.84	0.92	0.91	0.94	0.94	0.91	0.88	0.93	0.93	0.95	0.92	1.00	0.97	0.97	0.98	0.97	0.95	0.92	0.94	0.46	0.55	0.30	0.30	0.62	0.31	0.41	0.42	0.40
GK1028A	0.82	0.82	0.82	0.93	0.89	0.93	0.93	0.92	0.90	0.91	0.91	0.95	0.94	0.97	1.00	0.98	0.97	0.95	0.96	0.93	0.94	0.47	0.55	0.30	0.31	0.63	0.32	0.41	0.43	0.41
GK1029B	0.82	0.82	0.82	0.94	0.89	0.93	0.93	0.92	0.90	0.91	0.91	0.95	0.95	0.97	0.98	1.00	0.97	0.95	0.97	0.93	0.94	0.47	0.55	0.31	0.32	0.63	0.32	0.42	0.43	0.42
GK1030B	0.81	0.85	0.85	0.92	0.91	0.93	0.93	0.90	0.88	0.92	0.93	0.95	0.93	0.98	0.97	0.97	1.00	0.96	0.96	0.92	0.94	0.45	0.54	0.29	0.29	0.62	0.30	0.41	0.41	0.39
GK1034A2	0.79	0.85	0.85	0.90	0.91	0.93	0.93	0.88	0.85	0.93	0.93	0.94	0.90	0.97	0.95	0.95	0.96	1.00	0.94	0.91	0.93	0.45	0.54	0.28	0.29	0.61	0.31	0.39	0.38	0.36
GK1035A	0.81	0.85	0.83	0.92	0.90	0.91	0.91	0.91	0.90	0.91	0.90	0.94	0.95	0.95	0.96	0.97	0.96	0.94	1.00	0.92	0.94	0.46	0.54	0.29	0.31	0.62	0.32	0.41	0.42	0.40
CDC1121-73A_2	0.82	0.82	0.82	0.93	0.88	0.92	0.92	0.92	0.92	0.91	0.90	0.92	0.90	0.92	0.93	0.93	0.92	0.91	0.92	1.00	0.93	0.49	0.54	0.30	0.32	0.64	0.32	0.40	0.41	0.40
J172	0.82	0.85	0.84	0.92	0.90	0.92	0.93	0.91	0.90	0.92	0.92	0.93	0.91	0.94	0.94	0.94	0.94	0.93	0.94	0.93	1.00	0.47	0.55	0.31	0.33	0.62	0.34	0.41	0.42	0.40
Ctur3032	0.45	0.37	0.37	0.47	0.43	0.46	0.46	0.47	0.46	0.45	0.45	0.45	0.45	0.46	0.47	0.47	0.45	0.45	0.46	0.49	0.47	1.00	0.55	0.28	0.29	0.48	0.29	0.40	0.41	0.39
Cuni797-2	0.51	0.48	0.47	0.54	0.52	0.54	0.54	0.54	0.53	0.53	0.53	0.54	0.53	0.55	0.55	0.55	0.54	0.54	0.54	0.54	0.55	0.55	1.00	0.30	0.32	0.55	0.34	0.40	0.40	0.39
CconLMG26250	0.28	0.21	0.22	0.31	0.26	0.30	0.31	0.32	0.30	0.27	0.28	0.29	0.29	0.30	0.30	0.31	0.29	0.28	0.29	0.30	0.31	0.28	0.30	1.00	0.36	0.29	0.30	0.39	0.40	0.40
CdubCFS237	0.30	0.22	0.22	0.31	0.27	0.30	0.31	0.33	0.32	0.28	0.28	0.29	0.31	0.30	0.31	0.32	0.29	0.29	0.31	0.32	0.33	0.29	0.32	0.36	1.00	0.31	0.45	0.37	0.38	0.38
CmalCI825	0.58	0.52	0.52	0.62	0.58	0.61	0.61	0.62	0.61	0.60	0.60	0.62	0.62	0.62	0.63	0.63	0.62	0.61	0.62	0.64	0.62	0.48	0.55	0.29	0.31	1.00	0.32	0.40	0.42	0.40
Cmuy51329B	0.30	0.27	0.27	0.31	0.29	0.30	0.31	0.32	0.32	0.30	0.30	0.31	0.32	0.31	0.32	0.32	0.30	0.31	0.32	0.32	0.34	0.29	0.34	0.30	0.45	0.32	1.00	0.35	0.34	0.34
CQ118E. hormaechei	0.40	0.31	0.31	0.44	0.40	0.43	0.41	0.42	0.41	0.41	0.43	0.39	0.40	0.41	0.41	0.42	0.41	0.39	0.41	0.40	0.41	0.40	0.40	0.39	0.37	0.40	0.35	1.00	0.87	0.64
CQ119E.hormaechei	0.42	0.30	0.30	0.44	0.38	0.43	0.41	0.44	0.43	0.40	0.42	0.40	0.42	0.42	0.43	0.43	0.41	0.38	0.42	0.41	0.42	0.41	0.40	0.40	0.38	0.42	0.34	0.87	1.00	0.66
CQ125Citrosedlakii	0.40	0.27	0.27	0.44	0.37	0.42	0.39	0.43	0.42	0.38	0.39	0.39	0.40	0.40	0.41	0.42	0.39	0.36	0.40	0.40	0.40	0.39	0.39	0.40	0.38	0.40	0.34	0.64	0.66	1.00

*** Pearson's correlation analysis was performed as described by Tall *et al.* [15] using the Affymetrix algorithm. Cells highlighted in dark green represent a lower number of gene differences among the strains. Cells highlighted in red represent a higher number of gene differences.**
