# Supplementary material for: Genomic characterization of malonate positive Cronobacter sakazakii serotype O:2, sequence type 64 strains, isolated from clinical, food, and environment samples
Source: Gut Pathog. 2018 Mar 10;10:11. doi: 10.1186/s13099-018-0238-9 (PMC5845375; doi:10.1186/s13099-018-0238-9)
Supplement: Supplementary file 2 — Additional file 2: Table S2. Gene difference analysis of malonate-positive C. sakazakii strains compared to other Cronobacter and non Cronobacter strains. [file 13099_2018_238_MOESM2_ESM.pdf]

Supplemental Table 2. Gene difference anlaysis of malonate-positive *C. sakazakii* strains compared to other *Cronobacter* and non *Cronobacter* strains*.

Strains	CsakBAA894	Csak Comp11A	Csak Comp20A	Csak Comp53A	Csak Comp45B	Csak Comp46B	Csak Comp49B	Csak Comp54A	Csak Comp19B	Csak Comp55A	Csak Comp57A	Csak Comp59A	CDC1121-73A_2	E772	GK1025A	GK1027A	GK1028A	GK1029B	GK1030B	GK1034A2	GK1035A	J172	GK1036B2	Ctur3032	Cuni797-2	CconLMG26250	CdubCFS237	CmalCI825	Cmuy51329B	CQ118E. hormaeche	CQ119E.hormaechei	CQ125Citrosedlakii
CsakBAA894	0	657	648	662	674	652	603	635	629	649	648	621	620	621	592	616	613	613	618	615	599	597	684	2676	1986	3625	2658	1920	2721	1260	1241	1305
Csak Comp11A	657	0	0	0	159	157	129	115	119	14	16	169	179	184	156	164	164	165	160	165	153	219	497	2397	1683	3245	2358	1536	2387	1005	1022	1041
Csak Comp20A	648	0	0	0	156	162	125	102	105	11	10	163	171	175	148	158	152	154	153	153	154	201	471	2263	1604	3099	2178	1508	2251	926	949	974
Csak Comp53A	662	0	0	0	169	167	135	113	113	14	13	176	188	189	160	168	171	170	171	176	166	228	505	2397	1703	3266	2365	1586	2409	1052	1081	1099
Csak Comp45B	674	159	156	169	0	0	128	109	111	179	177	171	216	164	149	157	155	153	155	157	147	154	480	2365	1655	3151	2266	1575	2335	1098	1084	1147
Csak Comp46B	652	157	162	167	0	0	116	113	116	177	172	168	210	158	135	144	144	147	146	147	141	153	461	2305	1579	3095	2223	1507	2246	1004	967	1042
Csak Comp49B	603	129	125	135	128	116	0	37	42	121	120	159	136	156	143	155	152	152	150	154	144	145	415	2202	1494	3025	2196	1441	2193	946	882	969
Csak Comp54A	635	115	102	113	109	113	37	0	0	97	90	142	176	156	163	156	156	156	154	157	151	148	462	2296	1590	2983	2226	1550	2242	1054	1015	1106
Csak Comp19B	629	119	105	113	111	116	42	0	0	94	91	137	178	159	161	154	160	158	151	157	155	143	459	2230	1552	2961	2188	1542	2201	1041	999	1096
Csak Comp55A	649	14	11	14	179	177	121	97	94	0	0	163	171	172	171	183	182	183	182	183	175	221	489	2397	1736	3245	2404	1610	2430	1063	1087	1121
Csak Comp57A	648	16	10	13	177	172	120	90	91	0	0	156	170	173	169	180	174	174	177	178	168	213	488	2318	1680	3162	2318	1558	2343	1022	1032	1077
Csak Comp59A	621	169	163	176	171	168	159	142	137	163	156	0	182	157	148	158	155	159	155	158	154	177	455	2347	1691	3152	2312	1557	2337	1016	973	1085
CDC1121-73A_2	620	179	171	188	216	210	136	176	178	171	170	182	0	168	167	181	177	176	176	179	170	174	447	2243	1654	3216	2268	1456	2333	1074	1057	1116
E772	621	184	175	189	164	158	156	156	159	172	173	157	168	0	51	50	49	51	48	50	45	113	398	2397	1681	3251	2391	1513	2407	1069	1070	1136
GK1025A	592	156	148	160	149	135	143	163	161	171	169	148	167	51	0	0	0	0	0	0	0	121	391	2229	1546	3047	2222	1419	2251	945	904	977
GK1027A	616	164	158	168	157	144	155	156	154	183	180	158	181	50	0	0	0	0	0	0	0	120	406	2323	1626	3189	2277	1516	2341	1051	1049	1119
GK1028A	613	164	152	171	155	144	152	156	160	182	174	155	177	49	0	0	0	0	0	0	0	121	404	2324	1620	3202	2296	1480	2343	1032	1017	1078
GK1029B	613	165	154	170	153	147	152	156	158	183	174	159	176	51	0	0	0	0	0	0	0	125	405	2299	1601	3162	2246	1476	2303	1023	995	1073
GK1030B	618	160	153	171	155	146	150	154	151	182	177	155	176	48	0	0	0	0	0	0	0	116	409	2337	1631	3193	2294	1509	2326	1050	1047	1105
GK1034A2	615	165	153	176	157	147	154	157	157	183	178	158	179	50	0	0	0	0	0	0	0	120	407	2315	1637	3167	2259	1519	2348	1072	1052	1143
GK1035A	599	153	154	166	147	141	144	151	155	175	168	154	170	45	0	0	0	0	0	0	0	112	398	2297	1583	3150	2246	1469	2292	990	968	1026
J172	597	219	201	228	154	153	145	148	143	221	213	177	174	113	121	120	121	125	116	120	112	0	403	2227	1550	3029	2105	1481	2142	1025	1009	1065
GK1036B2	684	497	471	505	480	461	415	462	459	489	488	455	447	398	391	406	404	405	409	407	398	403	0	2372	1721	3252	2339	1581	2413	1085	1075	1156
Ctur3032	2676	2397	2263	2397	2365	2305	2202	2296	2230	2397	2318	2347	2243	2397	2229	2323	2324	2299	2337	2315	2297	2227	2372	0	2059	3932	2920	2420	2924	1361	1291	1416
Cuni797-2	1986	1683	1604	1703	1655	1579	1494	1590	1552	1736	1680	1691	1654	1681	1546	1626	1620	1601	1631	1637	1583	1550	1721	2059	0	3164	2185	1800	2217	1072	1032	1064
CconLMG26250	3625	3245	3099	3266	3151	3095	3025	2983	2961	3245	3162	3152	3216	3251	3047	3189	3202	3162	3193	3167	3150	3029	3252	3932	3164	0	3116	3465	3338	1790	1761	1804
CdubCFS237	2658	2358	2178	2365	2266	2223	2196	2226	2188	2404	2318	2312	2268	2391	2222	2277	2296	2246	2294	2259	2246	2105	2339	2920	2185	3116	0	2476	2208	1138	1102	1179
CmalCI825	1920	1536	1508	1586	1575	1507	1441	1550	1542	1610	1558	1557	1456	1513	1419	1516	1480	1476	1509	1519	1469	1481	1581	2420	1800	3465	2476	0	2557	1198	1158	1212
Cmuy51329B	2721	2387	2251	2409	2335	2246	2193	2242	2201	2430	2343	2337	2333	2407	2251	2341	2343	2303	2326	2348	2292	2142	2413	2924	2217	3338	2208	2557	0	1260	1221	1222
CQ118E. hormaeche	1260	1005	926	1052	1098	1004	946	1054	1041	1063	1022	1016	1074	1069	945	1051	1032	1023	1050	1072	990	1025	1085	1361	1072	1790	1138	1198	1260	0	63	111
CQ119E.hormaeche	1241	1022	949	1081	1084	967	882	1015	999	1087	1032	973	1057	1070	904	1049	1017	995	1047	1052	968	1009	1075	1291	1032	1761	1102	1158	1221	63	0	120
CQ125Citrosedlakii	1305	1041	974	1099	1147	1042	969	1106	1096	1121	1077	1085	1116	1136	977	1119	1078	1073	1105	1143	1026	1065	1156	1416	1064	1804	1179	1212	1222	111	120	0

*** Gene difference analysis was performed as described by Tall *et al.* [15] using the Affymetrix algorithm. Cells highlighted in dark green represent a lower number of gene differences among the strains. Cells highlighted in red represent a higher number of gene differences.**
