# Supplementary material for: Genomic characterization of malonate positive Cronobacter sakazakii serotype O:2, sequence type 64 strains, isolated from clinical, food, and environment samples
Source: Gut Pathog. 2018 Mar 10;10:11. doi: 10.1186/s13099-018-0238-9 (PMC5845375; doi:10.1186/s13099-018-0238-9)
Supplement: Supplementary file 4 — Additional file 4: Table S3. Description of malonate utilization operon in ST64 Cronobacter strains described in this study. [file 13099_2018_238_MOESM4_ESM.pdf]

Supplemental Table 3. Description of malonate utilization operon in ST64 *Cronobacter* strains described in this study.

	Malonate operon gene size in *Cronobacter* species (bp)																					
**Malonate utilization operon gene**	**Comp 11**	**Comp 19**	**Comp 20**	**Comp29**	**Comp46**	**Comp49**	**Comp53**	**Comp54**	**Comp55**	**Comp57**	**Comp59**	**GK 1025**	**GK 1026**	**GK 1027**	**GK 1029**	**GK 1030**	**GK 1034**	**GK 1035**	**GK 1326**	**E722**	**H169/1/16**	**Jor172**	**C.sak 112**
**Malonate decarboxylase alpha subunit**	1656	1656	1656	1656	1656	1656	1656	1656	1656	1656	1656	1656	1656	1656	1656	1656	1656	1656	1656	1656	1656	1656	1656
**Triphosphoribosyl-dephospho-CoA synthetase (EC 2.7.8.25)**	861	861	861	861	861	861	861	861	861	861	861	861	861	861	861	861	861	861	861	861	861	861	861
**Malonate decarboxylase delta subunit**	219	219	219	219	219	219	219	219	219	219	219	219	219	***300***	219	219	219	219	219	***300***	219	219	***300***
**Malonate decarboxylase beta subunit**	834	834	834	834	834	834	834	834	834	834	834	834	834	834	834	834	834	834	834	834	834	834	834
**Malonate decarboxylase gamma subunit**	801	801	801	801	801	801	801	801	801	801	801	801	801	801	801	801	801	801	801	801	801	801	801
**auxin efflux carrier / FIG00613710: hypothetical protein**	960	960	960	960	960	960	960	960	960	960	960	960	960	960	960	960	960	960	960	960	960	960	***603***
**Phosphoribosyl-dephospho-CoA transferase (EC 2.7.7.-)**	621	621	621	621	621	621	621	621	621	621	621	621	621	621	621	621	621	621	621	621	621	621	621
**Malonyl CoA acyl carrier protein transacylase (EC 2.3.1.39)**	903	903	903	903	903	903	903	903	903	903	903	903	903	903	903	903	903	903	903	903	903	903	903
**Malonate utilization transcriptional regulator**	930	930	930	930	930	930	930	930	930	930	930	930	930	930	930	930	930	930	930	930	930	930	930
**Total size of operon (bp)**	**7785**	**7785**	**7785**	**7785**	**7785**	**7785**	**7785**	**7785**	**7785**	**7785**	**7785**	**7785**	**7785**	**7866**	**7785**	**7785**	**7785**	**7785**	**7785**	**7866**	**7785**	**7785**	**7509**
