# Supplementary material for: Genomic characterization of malonate positive Cronobacter sakazakii serotype O:2, sequence type 64 strains, isolated from clinical, food, and environment samples
Source: Gut Pathog. 2018 Mar 10;10:11. doi: 10.1186/s13099-018-0238-9 (PMC5845375; doi:10.1186/s13099-018-0238-9)
Supplement: Supplementary file 5 — Additional file 5: Table S4. Additional table. [file 13099_2018_238_MOESM5_ESM.pdf]

Table 3.

Table 4. Microarray probe sets, probe set % similarities, NCBI protein and gene annotations, and hybridization results of corresponding to the malonate gene clusters of malonate-postive *Cronobacter* species including the malonate-positive *C. sakazakii* ST64 strains

C. sakzakii ST64 strains

	Probe	Probe species ID	ProbeSet	X_similarity	NCBI protein	NCBI annotations	BAA loci	BAA_neg	GR	core	CconLMG26250	CdubCFS237	Cmuy51329B	CmalCI825	Csak_Comp11A	Csak_Comp19B	Csak_Comp20A	Csak_Comp45B	Csak_Comp46B	Csak_Comp49B	Csak_Comp53A	Csak_Comp54A	Csak_Comp55A	Csak_Comp57A	Csak_Com	CDC1121	E772	I172	GK1025A	GK1027A	GK1028A	GK1029B	GK1030B	GK1034A'	GK1035A	GK1036B'	CsakBAA8	Ctur3032	Cuni797.1	CO118F_l	CO119F_b	CO125Citrosedlakii
4228	c_413498.3.1473_at	Cdubdub	1473	23.77	ABU77068	LysR family transcriptional regulator YneJ	ESA_01814	1629	GR34 - malonate		P*	P	A	A	A	A	A	A	A	A	A	A	A	A	A	A	A	A	A	A	A	A	A	A	A	A	A	A	A	A	A	A
4229	c_413498.3.1474_at	Cdubdub	1474	35.66	ABU77502	Malonyl CoA-acyl carrier protein transacylase;Ontology_term=KEGG_ENZYME:2.3.1.39	ESA_02253	2043	GR34 - malonate		P	P	A	A	A	A	A	A	A	A	A	A	A	A	A	A	A	A	A	A	A	A	A	A	A	A	A	A	A	A	A	A
4230	c_413498.3.1475_at	Cdubdub	1475	63.9	AEW61164	phosphoribosyl-dephospho-CoA_transferase		NA	GR34 - malonate		P	P	A	A	A	A	A	A	A	A	A	A	A	A	A	A	A	A	A	A	A	A	A	A	A	A	A	A	A	A	A	A
4231	c_413498.3.1476_at	Cdubdub	1476	87.77	ACI09216	malonate_transporter_MdcF;mdcF		NA	GR34 - malonate		P	P	A	A	A	A	A	A	A	A	A	A	A	A	A	A	A	A	A	A	A	A	A	A	A	A	A	A	A	A	A	A
4232	c_413498.3.1477_at	Cdubdub	1477	81.95	AEW61166	MdcE		NA	GR34 - malonate		A	P	A	A	A	A	A	A	A	A	A	A	A	A	A	A	A	A	A	A	A	A	A	A	A	A	A	A	A	A	A	A
4233	c_413498.3.1478_at	Cdubdub	1478	39.13	ABU76170	Acetyl-coenzyme A carboxyl transferase beta chain;Ontology_term=KEGG_ENZYME:6.4.1.2	ESA_00900	808	GR34 - malonate		P	P	A	A	A	A	A	A	A	A	A	A	A	A	A	A	A	A	A	A	A	A	A	A	A	A	A	A	A	A	A	A
4234	c_413498.3.1479_x_at	Cdubdub	1479	80.56	ACI11832	"malonate_decarboxylase	_delta_subunit;mdcC"	NA	GR34 - malonate		P	P	A	A	A	A	A	A	A	A	A	A	A	A	A	A	A	A	A	A	A	A	A	A	A	A	A	A	A	A	A	A
4235	c_413498.3.1480_at	Cdubdub	1480	33.85	ABU75626	2-(5''-triphosphoribosyl)-3'-dephosphocoenzyme-A synthase;Ontology_term=KEGG_ENZYME:2.7.8.25	ESA_00327	294	GR34 - malonate		P	P	A	A	A	A	A	A	A	A	A	A	A	A	A	A	A	A	A	A	A	A	A	A	A	A	A	A	A	A	A	A
4236	c_413498.3.1481_at	Cdubdub	1481	93.28	ACI08064	"malonate_decarboxylase	_alpha_subunit;mdcA"	NA	GR34 - malonate		P	P	A	A	A	A	A	A	A	A	A	A	A	A	A	A	A	A	A	A	A	A	A	A	A	A	A	A	A	A	A	A
9720	c_413501.3.3636_at	Cmuy	3636	22.67	ABU77068	LysR family transcriptional regulator YneJ	ESA_01814	1629	GR34 - malonate		A	A	P	A	A	A	A	A	A	A	A	A	A	A	A	A	A	A	A	A	A	A	A	A	A	A	A	A	A	A	A	A
9721	c_413501.3.3637_at	Cmuy	3637	37.6	ABU77502	Malonyl CoA-acyl carrier protein transacylase;Ontology_term=KEGG_ENZYME:2.3.1.39	ESA_02253	2043	GR34 - malonate		A	A	P	A	A	A	A	A	A	A	A	A	A	A	A	A	A	A	A	A	A	A	A	A	A	A	A	A	A	A	A	A
9722	c_413501.3.3638_at	Cmuy	3638	71.24	AEW61164	phosphoribosyl-dephospho-CoA_transferase		NA	GR34 - malonate		A	A	P	A	A	A	A	A	A	A	A	A	A	A	A	A	A	A	A	A	A	A	A	A	A	A	A	A	A	A	A	A
9723	c_413501.3.3639_at	Cmuy	3639	87.15	ACI09216	malonate_transporter_MdcF;mdcF		NA	GR34 - malonate		A	A	P	A	A	A	A	A	A	A	A	A	A	A	A	A	A	A	A	A	A	A	A	A	A	A	A	A	A	A	A	A
9724	c_413501.3.3640_at	Cmuy	3640	81.95	AEW61166	MdcE		NA	GR34 - malonate		A	A	A	A	A	A	A	A	A	A	A	A	A	A	A	A	A	A	A	A	A	A	A	A	A	A	A	A	A	A	A	A
9725	c_413501.3.3641_at	Cmuy	3641	36.65	ABU76170	Acetyl-coenzyme A carboxyl transferase beta chain;Ontology_term=KEGG_ENZYME:6.4.1.2	ESA_00900	808	GR34 - malonate		A	A	P	A	A	A	A	A	A	A	A	A	A	A	A	A	A	A	A	A	A	A	A	A	A	A	A	A	A	A	A	A
9726	c_413501.3.3642_x_at	Cmuy	3642	81.94	ACI11832	"malonate_decarboxylase	_delta_subunit;mdcC"	NA	GR34 - malonate		A	A	P	A	A	A	A	A	A	A	A	A	A	A	A	A	A	A	A	A	A	A	A	A	A	A	A	A	A	A	A	A
9727	c_413501.3.3643_at	Cmuy	3643	32.94	ABU75626	2-(5''-triphosphoribosyl)-3'-dephosphocoenzyme-A synthase;Ontology_term=KEGG_ENZYME:2.7.8.25	ESA_00327	294	GR34 - malonate		A	A	P	A	A	A	A	P	P	A	A	A	A	A	A	A	A	A	A	A	A	A	A	P	A	A	A	A	A	A	A	A
9728	c_413501.3.3644_at	Cmuy	3644	95.9	ACI08064	"malonate_decarboxylase	_alpha_subunit;mdcA"	NA	GR34 - malonate		A	A	P	A	A	A	A	A	A	A	A	A	A	A	A	A	A	A	A	A	A	A	A	A	A	A	A	A	A	A	A	A
10476	c_413503.3.1626_x_at	Cmal 825	1626	74.35	ACI08014	malonate_utilization_transcriptional_regulator_MdcR		NA	GR34		A	A	A	P	A	A	A	P	A	A	P	P	P	P	P	A	A	P	A	P	A	P	P	A	P	A	A	A	A	A	A	A
10478	c_413503.3.1627_at	Cmal 825	1627	37.6	ABU77502	Malonyl CoA-acyl carrier protein transacylase;Ontology_term=KEGG_ENZYME:2.3.1.39	ESA_02253	2043	GR34		A	A	A	P	P	P	P	P	P	P	P	P	P	P	P	P	P	P	P	P	P	P	P	P	P	A	A	A	A	A	A	A
10482	c_413503.3.1628_x_at	Cmal 825	1628	68.29	AEW61164	phosphoribosyl-dephospho-CoA_transferase		NA	GR34		A	A	A	A	A	A	A	A	A	A	A	A	A	A	A	A	A	A	A	A	A	A	A	A	A	A	A	A	A	A	A	A
10483	c_413503.3.1629_at	Cmal 825	1629	87.16	ACI09216	malonate_transporter_MdcF;mdcF		NA	GR34		A	A	A	P	P	A	P	P	P	A	P	A	P	P	P	P	P	P	P	P	P	P	P	P	P	A	A	A	A	A	A	A
10484	c_413503.3.1629_at	Cmal 825	1629	87.16	ACI09216	malonate_transporter_MdcF;mdcF		NA	GR34		A	A	A	P	P	A	P	P	P	A	P	A	P	P	P	P	P	P	P	P	P	P	P	P	P	A	A	A	A	A	A	A
10488	c_413503.3.1631_at	Cmal 825	1631	90.05	AEW61167	malonate_decarboxylase		NA	GR34		A	A	P	P	P	P	P	P	P	P	P	P	P	P	P	P	P	P	P	P	P	P	P	P	P	A	A	A	A	P	P	A
10489	c_413503.3.1632_x_at	Cmal 825	1632	87.5	ACI10965	"malonate_decarboxylase	_beta_subunit;mdcD"	NA	GR34		A	A	A	P	P	P	P	P	P	P	P	P	P	P	P	P	P	P	P	P	P	P	P	P	P	A	A	P	P	A	A	A
10490	c_413503.3.1633_x_at	Cmal 825	1633	79.17	ACI11832	"malonate_decarboxylase	_delta_subunit;mdcC"	NA	GR34		P	P	P	P	P	P	P	P	P	P	P	P	P	P	P	P	P	P	P	P	P	P	P	P	P	A	A	P	P	A	A	A
10491	c_413503.3.1634_x_at	Cmal 825	1634	32.68	ABU75626	2-(5''-triphosphoribosyl)-3'-dephosphocoenzyme-A synthase;Ontology_term=KEGG_ENZYME:2.7.8.25	ESA_00327	294	GR34		A	A	A	A	A	A	A	A	A	A	A	A	A	A	A	A	A	A	A	A	A	A	A	A	A	A	A	A	A	A	A	A
10492	c_413503.3.1635_at	Cmal 825	1635	96.09	ACI08064	"malonate_decarboxylase	_alpha_subunit;mdcA"	NA	GR34		A	A	A	P	P	A	P	P	P	P	P	P	P	P	P	P	P	P	P	P	P	P	P	P	P	A	A	A	A	A	A	A
10493	c_413503.3.1635_at	Cmal 825	1635	96.09	ACI08064	"malonate_decarboxylase	_alpha_subunit;mdcA"	NA	GR34		A	A	A	P	P	A	P	P	P	P	P	P	P	P	P	P	P	P	P	P	P	P	P	P	P	A	A	A	A	A	A	A
12040	c_535744.3.1165_s_at	Cuni	1165	93.83	ACI08064	"malonate_decarboxylase	_alpha_subunit;mdcA"	NA	GR34 - malonate		A	A	A	P	P	P	P	P	P	P	P	P	P	P	P	P	P	P	P	P	P	P	P	P	P	A	A	A	P	A	A	A
12043	c_535744.3.1166_s_at	Cuni	1166	32.68	ABU75626	2-(5''-triphosphoribosyl)-3'-dephosphocoenzyme-A synthase;Ontology_term=KEGG_ENZYME:2.7.8.25	ESA_00327	294	GR34 - malonate		A	A	A	P	P	P	P	P	P	P	P	P	P	P	P	P	P	P	P	P	P	P	P	P	P	A	A	A	P	A	A	A
12044	c_535744.3.1166_s_at	Cuni	1166	32.68	ABU75626	2-(5''-triphosphoribosyl)-3'-dephosphocoenzyme-A synthase;Ontology_term=KEGG_ENZYME:2.7.8.25	ESA_00327	294	GR34 - malonate		A	A	A	P	P	P	P	P	P	P	P	P	P	P	P	P	P	P	P	P	P	P	P	P	P	A	A	A	P	A	A	A
12045	c_535744.3.1167_at	Cuni	1167	83.84	AEW61168	malonate_decarboxylase_delta_subunit		NA	GR34 - malonate		P	P	P	P	A	P	P	P	P	A	A	P	P	P	P	P	P	P	P	P	P	P	P	P	P	A	A	A	P	A	A	A
12046	c_535744.3.1169_s_at	Cuni	1169	83.46	AEW61166	MdcE		NA	GR34 - malonate		A	A	P	P	A	P	P	P	A	P	P	P	P	P	P	P	P	P	A	P	P	P	P	P	P	A	A	A	P	A	A	A
12048	c_535744.3.1170_s_at	Cuni	1170	87.16	ACI09216	malonate_transporter_MdcF;mdcF		NA	GR34 - malonate		A	A	A	P	P	P	P	P	P	P	P	P	P	P	P	P	P	P	P	P	P	P	P	P	P	A	A	A	P	A	A	A
12049	c_535744.3.1171_at	Cuni	1171	68.29	AEW61164	phosphoribosyl-dephospho-CoA_transferase		NA	GR34 - malonate		A	A	A	A	A	A	A	A	A	A	A	A	A	A	A	A	A	A	A	A	A	A	A	A	A	A	A	A	A	A	A	A
12050	c_535744.3.1171_at	Cuni	1171	68.29	AEW61164	phosphoribosyl-dephospho-CoA_transferase		NA	GR34 - malonate		A	A	A	A	A	A	A	A	A	A	A	A	A	A	A	A	A	A	A	A	A	A	A	A	A	A	A	A	A	A	A	A
12053	c_535744.3.1172_at	Cuni	1172	36.82	ABU77502	Malonyl CoA-acyl carrier protein transacylase;Ontology_term=KEGG_ENZYME:2.3.1.39	ESA_02253	2043	GR34 - malonate		A	A	A	A	A	A	A	A	A	A	A	A	A	A	A	A	A	A	A	A	A	A	A	A	A	A	A	A	A	A	A	A
12054	c_535744.3.1173_s_at	Cuni	1173	74.84	ACI08014	malonate_utilization_transcriptional_regulator_MdcR		NA	GR34 - malonate		A	A	A	P	P	P	P	P	P	P	P	P	P	P	P	P	P	P	P	P	P	P	P	P	P	A	A	A	P	A	A	A
19105	c_6666.1845.3619_at	Ctur	3619	74.35	ACI08014	malonate_utilization_transcriptional_regulator_MdcR		NA	GR34 - malonate		A	A	A	A	A	A	A	A	A	A	A	A	A	A	A	A	A	A	A	A	A	A	A	A	A	A	A	P	A	A	A	A
19110	c_6666.1845.3620_at	Ctur	3620	37.21	ABU77502	Malonyl CoA-acyl carrier protein transacylase;Ontology_term=KEGG_ENZYME:2.3.1.39	ESA_02253	2043	GR34 - malonate		A	A	A	A	A	A	A	A	A	A	A	A	A	A	A	A	A	A	A	A	A	A	A	A	A	A	A	P	A	A	A	A
19111	c_6666.1845.3621_at	Ctur	3621	68.29	AEW61164	phosphoribosyl-dephospho-CoA_transferase		NA	GR34 - malonate		A	A	A	A	A	A	A	A	A	A	A	A	A	A	A	A	A	A	A	A	A	A	A	A	A	A	A	P	A	A	A	A
19112	c_6666.1845.3622_at	Ctur	3622	87.5	ACI09216	malonate_transporter_MdcF;mdcF		NA	GR34 - malonate		A	A	A	A	A	A	A	A	A	A	A	A	A	A	A	A	A	A	A	A	A	A	A	A	A	A	A	P	A	A	A	A
19113	c_6666.1845.3623_at	Ctur	3623	82.71	AEW61166	MdcE		NA	GR34 - malonate		A	A	A	A	A	A	A	A	A	A	A	A	A	A	A	A	A	A	A	A	A	A	A	A	A	A	A	P	A	A	A	A
19116	c_6666.1845.3624_s_at	Ctur	3624	36.13	ABU76170	Acetyl-coenzyme A carboxyl transferase beta chain;Ontology_term=KEGG_ENZYME:6.4.1.2	ESA_00900	808	GR34 - malonate		A	A	A	P	P	P	P	P	P	P	P	P	P	P	P	P	P	P	P	P	P	P	P	P	P	A	A	P	P	A	A	A
19117	c_6666.1845.3624_s_at	Ctur	3624	36.13	ABU76170	Acetyl-coenzyme A carboxyl transferase beta chain;Ontology_term=KEGG_ENZYME:6.4.1.2	ESA_00900	808	GR34 - malonate		A	A	A	P	P	P	P	P	P	P	P	P	P	P	P	P	P	P	P	P	P	P	P	P	P	A	A	P	P	A	A	A
19118	c_6666.1845.3625_x_at	Ctur	3625	80.56	ACI11832	"malonate_decarboxylase	_delta_subunit;mdcC"	NA	GR34 - malonate		P	P	P	P	P	P	P	P	P	P	A	P	P	P	P	P	P	P	P	P	A	P	P	P	P	A	A	P	P	A	A	A
19119	c_6666.1845.3626_at	Ctur	3626	33.85	ABU75626	2-(5''-triphosphoribosyl)-3'-dephosphocoenzyme-A synthase;Ontology_term=KEGG_ENZYME:2.7.8.25	ESA_00327	294	GR34 - malonate		A	A	A	A	A	A	A	A	A	A	A	A	A	A	A	A	A	A	A	A	A	A	A	A	A	A	A	A	A	A	A	A
19120	c_6666.1845.3627_at	Ctur	3627	93.65	ACI08064	"malonate_decarboxylase	_alpha_subunit;mdcA"	NA	GR34 - malonate		A	A	A	A	A	A	A	A	A	A	A	A	A	A	A	A	A	A	A	A	A	A	A	A	A	A	A	A	A	A	A	A

*** P represents presence and A represents absence of the allele. Presence/absence calls were determined using the Affymetrix algorithm as described by Tall et al. [15].**
