# Supplementary figures and images for: Genomic characterization of malonate positive Cronobacter sakazakii serotype O:2, sequence type 64 strains, isolated from clinical, food, and environment samples
Source: Gut Pathog. 2018 Mar 10;10:11. doi: 10.1186/s13099-018-0238-9 (PMC5845375; doi:10.1186/s13099-018-0238-9)

**Figure S2.**
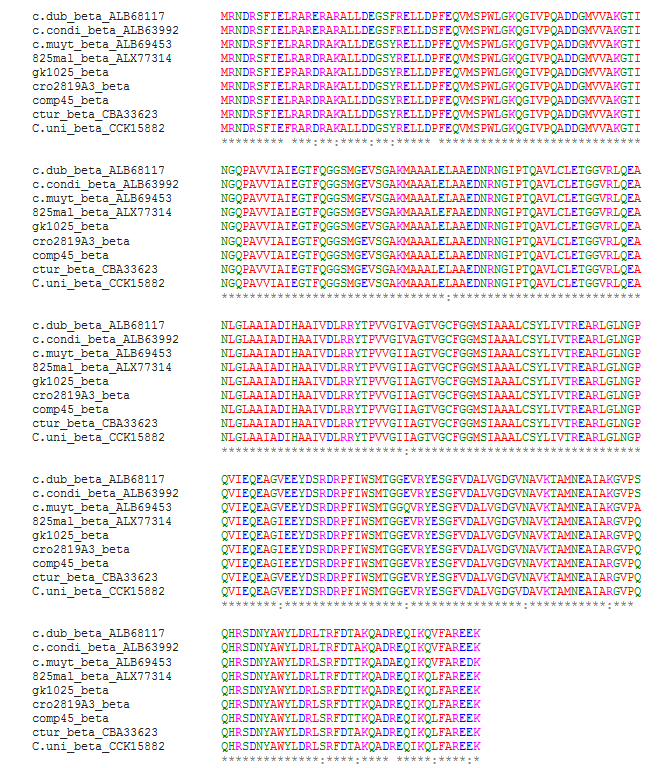

Supplement: Supplementary file 6 — Additional file 6: Figure S2. Clustal analysis of mdcB protein from ST64 and non-C. sakazakii strains. Proteins sequences were retrieved from the annotations found in NCBI or RAST/SEED server originally carried out as part of this study. The sequences were subjected Clustal Omega multiple alignment. Amino acid substitutions in different species are noted in the illustration reflecting the nucleotide diversity discussed elsewhere. [file 13099_2018_238_MOESM6_ESM.docx]
